# Extrahepatic cholangiography in near-infrared II window with the clinically approved fluorescence agent indocyanine green: a promising imaging technology for intraoperative diagnosis

**DOI:** 10.7150/thno.41127

**Published:** 2020-02-19

**Authors:** Di Wu, Dingwei Xue, Jing Zhou, Yifan Wang, Zhe Feng, Junjie Xu, Hui Lin, Jun Qian, Xiujun Cai

**Affiliations:** 1 Department of General Surgery, Sir Run-Run Shaw Hospital, School of Medicine, Zhejiang University, Hangzhou, 310016, China.; 2 State Key Laboratory of Modern Optical Instrumentations, Centre for Optical and Electromagnetic Research, College of Optical Science and Engineering, Zhejiang University, Hangzhou, 310058, China.; 3 Zhejiang Provincial Key Laboratory of Laparoscopic Technology, Hangzhou, 310016, China.; 4 Department of Urology, Sir Run-Run Shaw Hospital, School of Medicine, Zhejiang University, Hangzhou, 310016, China.

**Keywords:** bile duct injury, diagnosis, near-infrared II cholangiography, indocyanine green, fluorescence-guided surgery

## Abstract

**Rationale**: Biliary tract injury remains the most dreaded complication during laparoscopic cholecystectomy. New intraoperative guidance technologies, including near-infrared (NIR) fluorescence cholangiography with indocyanine green (ICG), are under comprehensive evaluation. Previous studies had shown the limitations of traditional NIR light (NIR-I, 700-900 nm) in visualizing the biliary tract structures in specific clinical situations. The aim of this study was to evaluate the feasibility of performing the extrahepatic cholangiography in the second NIR window (NIR-II, 900-1700 nm) and compare it to the conventional NIR-I imaging.

**Methods**: The absorption and emission spectra, as well as fluorescence intensity and photostability of ICG-bile solution in the NIR-II window were recorded and measured. *In vitro* intralipid^®^ phantom imaging was performed to evaluate tissue penetrating depth in NIR-I and NIR-II window. Different clinical scenarios were modeled by broadening the penetration distance or generating bile duct injuries, and bile duct visualization and lesion site diagnosis in the NIR-II window were evaluated and compared with NIR-I imaging.

**Results**: The fluorescence spectrum of ICG-bile solution extends well into the NIR-II region, exhibiting intense emission value and excellent photostability sufficient for NIR-II biliary tract imaging. Extrahepatic cholangiography using ICG in the NIR-II window obviously reduced background signal and enhanced penetration depth, providing more structural information and improved visualization of the bile duct or lesion location in simulated clinical scenarios, outperforming the NIR-I window imaging.

**Conclusions**: The conventional clinically approved agent ICG is an excellent fluorophore for NIR-II bile duct imaging. Fluorescence cholangiography with ICG in the NIR-II window could provide adequate visualization of the biliary tract structures with increased resolution and penetration depth and might be a valid option to increase the safety of cholecystectomy in difficult cases.

## Introduction

Iatrogenic biliary tract injuries remain the primary concern of general surgeons performing cholecystectomies, with an incidence ranging from 0.08% to 0.4% 1-3. The presence of acute cholecystitis could increase the rate of bile duct injuries by 20-fold 4 resulting in bile leakage or biliary tract occlusion with obstructive jaundice, which might eventually lead to liver transplantation in worst cases 5. Anatomical variations in the extrahepatic biliary tract, combined with inflammation or obesity or the surgeon misunderstanding the anatomy are among the reasons for the complications 6. Therefore, techniques to improve the identification of the anatomical structures are urgently desired.

There are mainly two intraoperative techniques to consider, one is the conventional X-ray based intraoperative cholangiography (IOC), and secondly the emerging fluorescence-based NIR extrahepatic cholangiography. Although IOC reduces the rate of bile duct injuries 7, 8, there are several drawbacks including radiation exposure, time-consuming, expertise dependence and manpower requirement, making it not adopted worldwide in standard cholecystectomy. On the other hand, NIR fluorescence imaging by intravenous injection of ICG is a promising technique for intraoperative cholangiography. In a recently published prospective, randomized multicenter trial, NIR fluorescence cholangiography was shown to be statistically superior to white light alone in visualizing extrahepatic biliary structures during laparoscopic cholecystectomy 9.

However, conventional fluorescent cholangiography also has some inherent deficiencies. The limited tissue penetration capability of NIR-I light and high autofluorescence background in this window could result in the inability to visualize the extrahepatic duct under special clinical conditions, such as obesity and cholecystitis 10. Moreover, commercial fluorescence imaging systems widely used in clinical research apply silicon-based charge-coupled device (CCD) or complementary metal-oxide-semiconductor (CMOS) cameras, the detection efficiency of which sharply declines beyond 900 nm 11, thereby hampering the further clinical application.

Recent studies in fluorescence imaging in the NIR-II window have shown significant advantages over NIR-I imaging 12-15. Reduced photon scattering effect in tissue components makes it possible to penetrate deeper into the underlying tissues 16-21. Therefore, performing fluorescence cholangiography in the NIR-II window might enable larger imaging depth and higher spatial resolution, providing more anatomical information even in severe cholecystitis or obesity.

Here, we applied the clinically approved agent ICG as a fluorophore for NIR-II extrahepatic cholangiography. The emission properties of ICG-bile solution in the NIR-II window were measured and compared to those of its aqueous solution. *In vitro* evaluation of tissue penetrating capability in the NIR-II window was carried out by imaging capillary tubes submerged in 1% Intralipid^®^ solution. Established rat or mouse models were used to perform *in vivo* comparison of the outcomes of NIR-I and NIR-II cholangiography. Also, measurement of the NIR-II emission properties and *in vitro* fluorescence intensity analysis were also performed in ICG-human bile solution to assess the clinical translation potentiality. The primary objective of the present study was to evaluate the validity and feasibility of applying the NIR-II imaging technique in extrahepatic cholangiography using a clinically approved fluorescence agent and demonstrate its strong potential for translation into clinical applications.

## Materials and Methods

The fluorophore ICG was purchased from DanDong Pharmaceutical Factory (Liaoning, China) and used without any further modification. Phosphate buffered saline (PBS) was obtained from Sinopharm Chemical Reagent Company (Hangzhou, China) and 20% intralipid^®^ was purchased from Baxter Healthcare Corporation (USA). Coomassie Brilliant Blue Fast Staining solution was purchased from Solaribo Life Sciences Company (P1300, Beijing, China). Pierce™ BCA Protein Assay Kit was acquired from Thermo Scientific Corporation (23227, Massachusetts, USA). Deionized (DI) water with the resistivity of 18.2 MΩ/cm was used in all experiments.

### Absorption and fluorescence emission characterization

Measurement of absorption spectra of ICG dilutions in bile and water were obtained from 550-900 nm utilizing a Shimadzu UV-2550 UV-vis-NIR scanning spectrophotometer. The fluorescence emission spectra of ICG dilutions in bile and water in the NIR-II window were collected by a lab-built system based on a PG2000 spectrometer (Ideaoptics Instruments) and a 2000C spectrometer (Everuping Optics Corporation).

### Absolute photoluminescence quantum yield measurement

The fluorescence quantum yield of ICG dilutions in water and bile were measured by Quantaurus-QY Plus (UV-NIR absolute photoluminescence quantum yield spectrometer C13534-31 with additional NIR photoluminescence measurement unit C13684-01, Hamamatsu, Japan), using an 808-nm laser as an excitation source. The samples to be measured were diluted and adjusted to proper concentration, the final optical density value of which ranged between 0.2-0.5 at 808-nm. The quantum yield of the sample was calculated by the following equation:


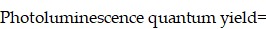



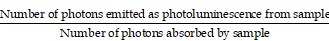


### NIR-II fluorescence imaging

As shown in Figure S1, a 2D electronic-cooling InGaAs camera (640 pixels×512 pixels, TEKWIN SYSTEM, China) equipped with a prime lens (focal length: 50 mm, antireflection coating at 800-2000 nm, Edmund Optics), cooled to -40℃, was used to acquire images in the NIR-II window. A 793-nm laser (Suzhou Rugkuta Optoelectronics Co., Ltd, China) beam was coupled to a collimator and expanded by a lens to provide uniform illumination on the field. The facular power density was measured before each imaging experiment and was adjusted to 10-20 mW/cm^2^. During imaging, two 800-nm long-pass filters were used to filter away 793 nm excitation. The various long-pass filters were utilized to extract different NIR-II fluorescence signals as required. For instance, a 1000-nm long-pass filter (ThorLabs, USA) was placed in front of the camera lens restricting wavelength below as well as allowing wavelength above 1000 nm to pass through the camera lens.

### NIR-I fluorescence imaging

Images in the NIR-I window were captured using a silicon camera (512 pixels×512 pixels, iXon Ultra 897, Andor, USA) equipped with a prime lens (focal length: 50 mm, antireflection coating at 800-2000 nm, Edmund Optics), which was fitted with two 800-nm long-pass filters and a 900-nm short pass filter to extract NIR-I fluorescence signal. A 793-nm laser beam was coupled to a collimator and expanded by a lens to provide uniform illumination on the field. The facular power density was adjusted to 10-20 mW/cm^2^.

### *In vivo* sub-surface extrahepatic bile duct imaging (n = 9)

After anesthetization, the rat was secured to a platform in the supine position, then laparotomy was performed, and the surgical field of interest was fully exposed. 0.5 mg/kg of ICG was intravenously injected through tail vein, using a 793-nm excitation (power intensity, 10 mW/cm^2^). NIR-I and NIR-II imaging were performed to visualize the extrahepatic bile duct structure. Then a piece of abdominal tissue was removed integrally from an anesthetized ICR mouse and instantly placed on the observing field of interest without any fold. NIR-I and NIR-II imaging were further performed to visualize the biliary structure through the covered tissue.

Moreover, fresh porcine adipose tissue or inflammatory abdominal tissue generated from an acute peritonitis mouse model was also acquired and covered on the region the bile duct went in rat models (n = 10, n = 9, respectively) for further comparison between the imaging performed in the NIR-I and NIR-II windows.

### Establishment of the acute bile duct stricture model (n = 6)

An acute bile duct stricture model was established by common bile duct ligation in the rat. In brief, after laparotomy, the common bile duct was partially separated and occluded by using a 5-0 suture, imitating intraoperative accident of bile duct ligation. ICG at a concentration of 0.5 mg/kg was intravenously injected through tail vein instantly after ligation, and the facular power density was adjusted to 10 mW/cm^2^. The rat was then placed in a supine position under the NIR-I or NIR-II camera to visualize the extrahepatic bile duct and detect the ligation point. An intact piece of abdominal tissue (approximate 1.2 mm thickness) was removed from an anesthetized ICR mouse and instantly placed superficially on the surgical field of interest without any fold. Subsequently, NIR-I and NIR-II imaging were performed as previously described to detect the precise bile duct lesion location through the covered tissue.

### Establishment of the acute biliary injury models

An acute biliary perforation model (n = 6) was established as previously reported 22. After laparotomy, ICG at a concentration of 0.5 mg/kg was administrated through tail vein of the rat. When the fluorophore was excreted into extrahepatic biliary system 5 minutes post-injection, a microsurgical scissor was used to perforate the common bile duct. Then the rat was immediately placed in a supine position under NIR-II detector to visualize the leakage of bile from the damaged common bile duct.

An acute complete common bile duct transection model (n = 6) was established by transecting the rat common bile duct with electrotome (GD350-B, HuTong electronics Co., Ltd, China), both proximal and distal end of which would be occluded. Then NIR-I and NIR-II imaging were performed to detect the transection site. Moreover, a piece of fresh abdominal tissue was placed superficially on the surgical field of interest and NIR-I and NIR-II imaging were further performed to detect the precise lesion location through the covering tissue.

### Ethical approval

All animal experiments in this study were conducted strictly in compliance with the requirements and guidelines of the Institutional Ethical Committee of Animal Experimentation of Zhejiang University. All procedures performed in studies involving human participants were in accordance with the ethical standards of the Clinical Research Ethics Committee of Sir Run-Run Shaw Hospital of Zhejiang University.

The detailed experimental materials and methods are listed in supplementary information.

### Data analysis

Quantitative analysis of each fluorescent image was performed based on the measurement of mean signal intensity in the manually selected regions of interest, using Image J software (Version 1.6.0, National Institutes of Health, USA). Graphs were generated using Origin Pro software (Version 9.0, OriginLab Company, USA). The data were presented as mean ± standard deviation (SD). Statistical analysis was performed using Student's t-test. * denotes a statistical significance (* P < 0.05, ** P < 0.01, and *** P < 0.001) between the experimental data of two groups.

## Results

### Absorption and fluorescence emission spectra

**Figure 1:**
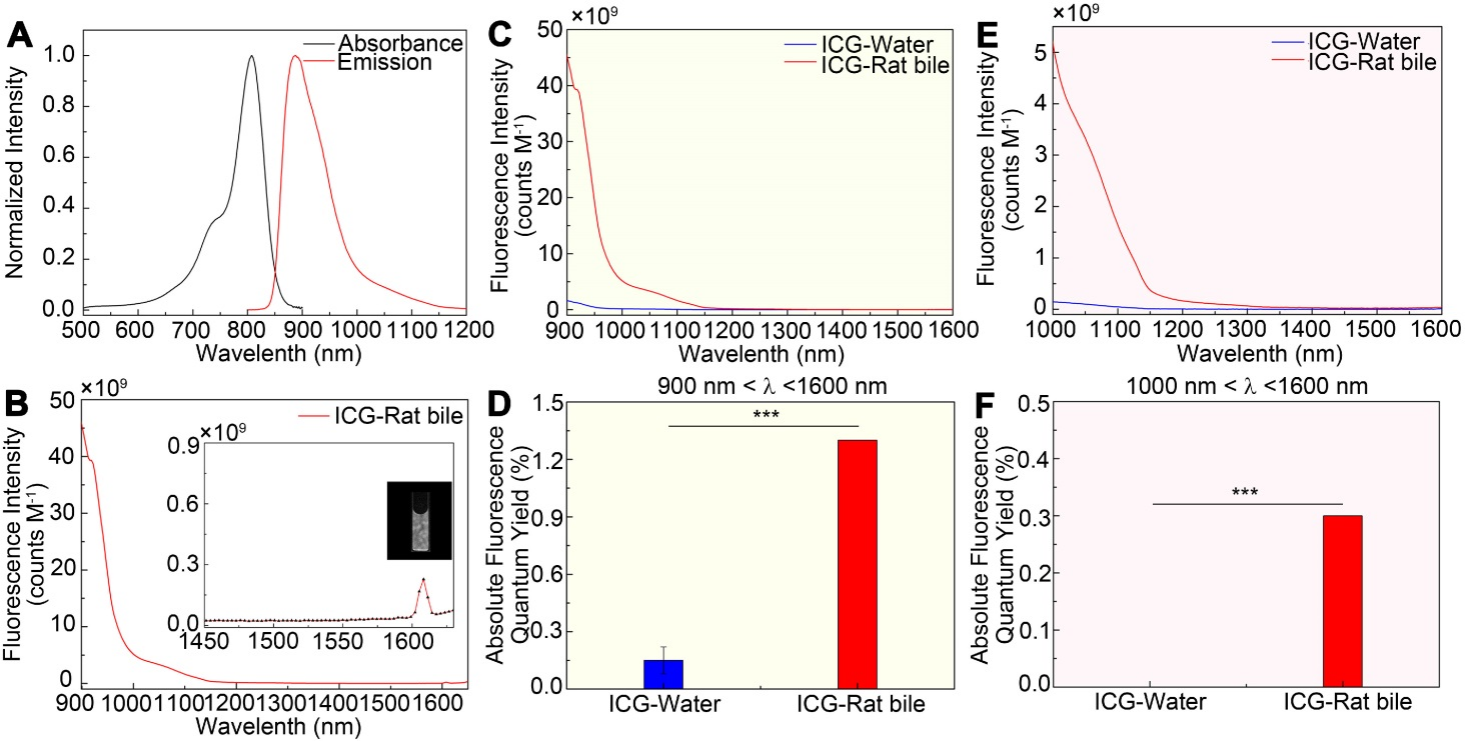
**NIR-II emission of ICG-rat bile solution.** (A) Absorbance and emission spectrum of ICG-rat bile solution. (B) Emission fluorescence intensity of ICG-rat bile solution normalized to equimolar concentration in the NIR-II window. The emission is detectable up to at least 1600 nm (Inset shows the vial). Fluorescence emission profile of ICG-rat bile and ICG-water solution normalized to an equimolar concentration between (C) 900 and 1600 nm wavelength region, (E) 1000 and 1600 nm wavelength region. Absolute fluorescence quantum yield measurement of ICG-rat bile solution and ICG-water solution between (D) 900 and 1600 nm wavelength region, (F) 1000 and 1600 nm wavelength region.

The emission spectrum of the ICG-rat bile solution was recorded on a system sensitive to both NIR-I and NIR-II light (detected by the PG2000 spectrometer and 2000C spectrometer respectively), showing that the ICG's fluorescence emission extended well into the NIR-II region (Figure 1A-B). Furthermore, the fluorescence emission could be detected up to at least 1600-nm wavelength (Inset shows the vial), using 793-nm excitation (Figure 1B).

The fluorescence emission spectra of ICG-rat bile and ICG-water solutions were determined and compared over 900 to 1600 nm wavelength range. In aqueous solution, ICG exhibited a relatively low fluorescence emission value, whereas after being excreted into rat bile, the fluorescence intensity in different NIR-II spectral ranges was much greater than that of ICG in aqueous solution (Figure 1C, E). This finding was also confirmed by calculating the absolute quantum yield (QY) of ICG dilutions in rat bile and water in corresponding NIR-II regions (Figure 1D, F). At wavelengths between 900-1600 nm, the QY of ICG-rat bile was measured to be 1.3%, nearly 13 times higher than that (0.1%) of ICG-water solution. At wavelengths between 1000-1600 nm, the QY of ICG-rat bile solution was 0.3%, whereas the QY of ICG-water solution could barely be measured. For ICG dilution in human bile, the full absorbance and NIR-II emission spectrum, as well as QY in the NIR-II window were measured and recorded, which also extended well into NIR-II region and exhibited higher QY in the NIR-II window (Figure S3A-F).

### *In vitro* fluorescence intensity phantom imaging

**Figure 2:**
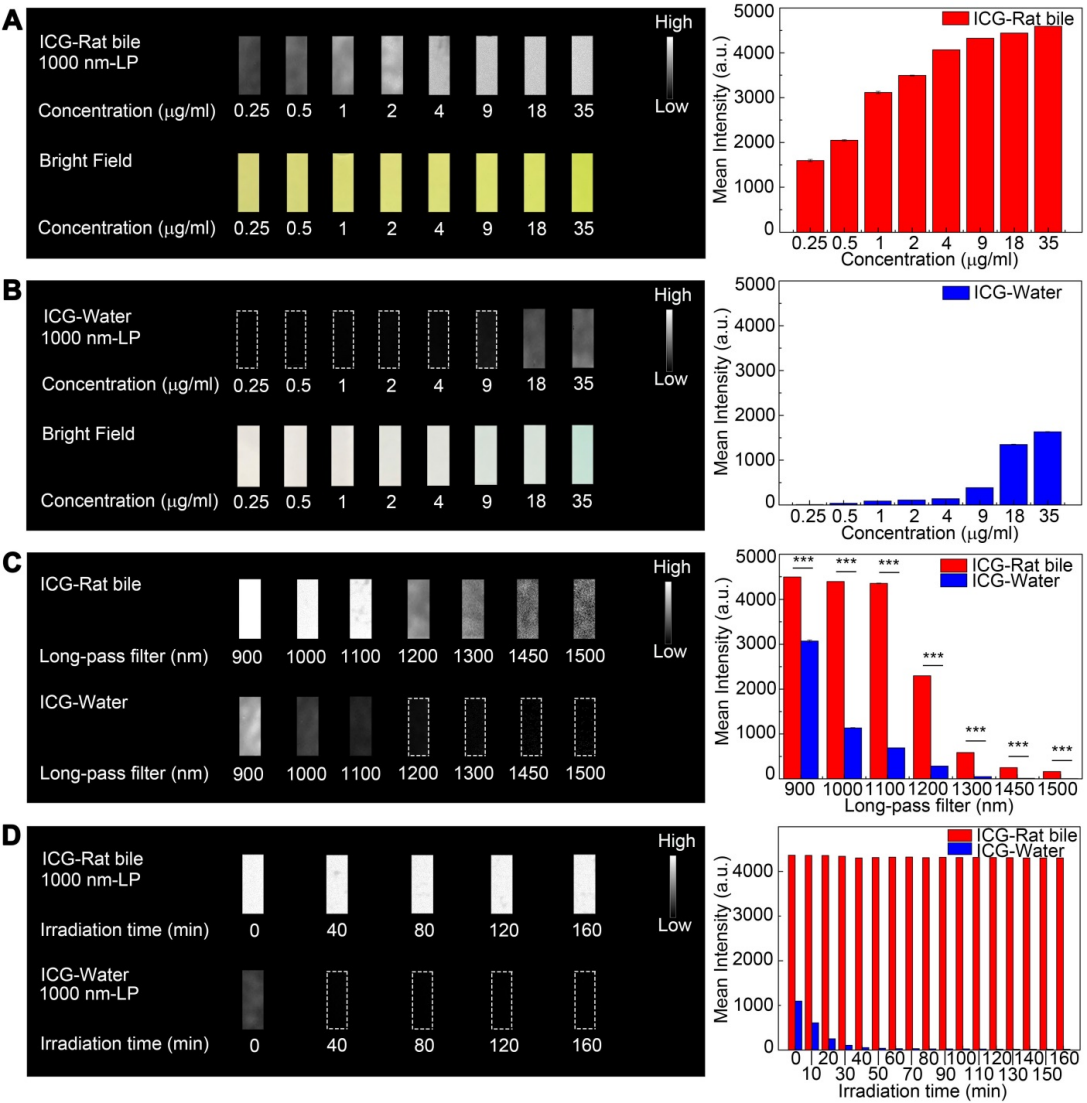
**Comparison of optical properties of ICG-rat bile and ICG-water solution in NIR-II window.** (A) NIR-II fluorescence and bright field images of vials filled with (A, left) ICG-rat bile solution and (B, left) ICG-water solution at 8 different concentrations. Variations of ICG NIR-II fluorescence intensity measurement as a function of its concentration (μg/ml) in (A, right) rat bile and (B, right) water. (C, left) NIR-II Fluorescence images of vials filled with ICG-rat bile solution and ICG-water solution (both 35μg/ml) in different wavelength regions. (C, right) NIR-II fluorescence intensity measurement of ICG-rat bile and water solution at concentration of 35 μg/ml with 900-, 1000-, 1100-, 1200-, 1300-, 1450-, 1500-nm long-pass filter. (D, left) Photostability of ICG-rat bile solution and ICG-water solution under the excitation of a 793-nm laser. Insets show NIR-II fluorescence photos of the two kinds of solution at each time point during laser illumination, power density, 45 mW/cm^2^. (D, right) NIR-II fluorescence intensity measurement of ICG-rat bile solution and ICG-water solution at each time point during laser illumination.

At each equivalent concentration, the ICG-rat bile solution emitted a more intense fluorescence signal compared to its aqueous solution (Figure 2A-B). The NIR-II emission intensity of ICG dilutions in pure water and rat bile at 35 μg/ml were also investigated and compared under wavelengths above 900-, 1000-, 1100-, 1200-, 1300-, 1450- and 1500-nm. Only extremely weak signal from aqueous ICG solution could be detected with an 1100-nm long-pass filter (Figure 2C). On the other hand, the fluorescence signal of bilious solution at each wavelength region was much stronger and could be detected even with a 1500-nm long-pass filter by properly increasing the exposure time to 100 ms. We also performed NIR-II fluorescence intensity analysis in the ICG-human bile solution, which showed emission of intense fluorescence signal in the NIR-II window (Figure S4A-C).

### Photostability testing

The emission intensity of ICG-rat bile solution in the NIR-II window decreased by ~1.4% after continuous illumination from 793 nm laser with a power density of 45 mW/cm^2^ for 160 minutes, the ICG-rat bile solution could still emit strong fluorescence signal at short integration time (5ms), exhibiting better photostability compared to that of aqueous solution (Figure 2D). Similar testing was also performed on ICG-human bile solution, which also showed good photostability (Figure S4D).

### Coomassie Brilliant Blue staining on bile

Coomassie Brilliant Blue staining showed that the fresh bile contained a large number of protein components ranging from 10 to 250 kilodalton (kDa). The distribution of the proteins in rat bile varied. As shown in Figure S5A, protein compositions were similar in samples 1 and 2, mainly ranging between 25 and 55 kDa; for sample 3, the most proteins were between 65 and 85 kDa. We also performed Coomassie Brilliant Blue staining of human bile and found that the proteins mainly ranged between 25 and 75 kDa, of which about 65 kDa showed the highest concentration (Figure S5B). Despite the heterogeneity, the staining results provided strong evidence that the bile contained abundant protein components, which could provide various potential binding sites.

### Intralipid^®^ phantom imaging

**Figure 3:**
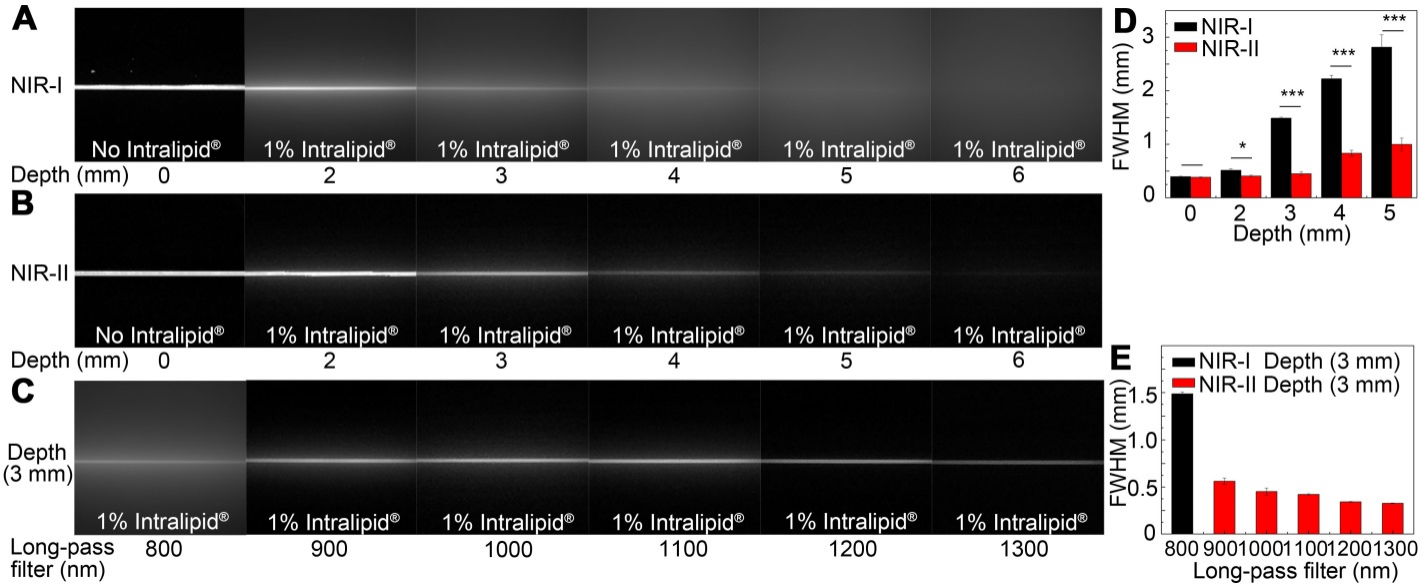
**Intralipid^®^ phantom study of ICG-bile solution in NIR-I and NIR-II window.** Fluorescence images in (A) NIR-I window and (B) NIR-II window of glass capillary filled with ICG-rat bile solution (31.25 μg/ml) at depths of 0, 2, 3, 4, 5, and 6 mm in 1% Intralipid^®^ solution. (C) The capillary filled with ICG-rat bile solution was submerged in 3 mm of 1% intralipid^®^ solution and imaged with 800-nm long-pass NIR-I detector and 900-, 1000-, 1100-, 1200-, and 1300-nm long-pass NIR-II detector. FWHM was calculated for a capillary glass tube filled with ICG-rat bile solution (D) at varying depths of 1% intralipid^®^ solution or (E) with different long-pass filters at the same depth of 1% intralipid^®^ solution.

A tissue phantom study using Intralipid^®^ mimicking the optical characteristics of biological tissues was performed to compare the penetration depth of ICG-rat bile solution between the NIR-I and NIR-II windows. When the 1% Intralipid^®^ solution was not added, sharp images could be formed in both NIR-I and NIR-II windows. However, the scattering effect became significantly pronounced with the increasing depth in the NIR-I window (Figure 3A). By contrast, sharp edges of capillary tubes could be maintained in the NIR-II window even at a penetration depth of 5 mm (Figure 3B). A full-width-half-maximum (FWHM) analysis depicting the feature width of NIR-I and NIR-II capillary images at varying depths in intralipid^®^ phantom assay was also plotted (Figure 3D). The FWHM measurements of the capillary tube without 1% Intralipid^®^ solution were 394.4±13.3 μm and 383.5±6.3 μm in the NIR-I and NIR-II window, respectively. When the depth increased to 5 mm, the FWHM measured 2813.9±234.6 μm and 994.9±13.3 μm in the NIR-I and NIR-II windows, respectively. The width of the capillary tube was significantly broadened with the increase of penetration depth in the NIR-I window, but not in the NIR-II window. Next, the capillary tube filled with ICG-rat bile solution submerged in 1% intralipid^®^ liquid at a depth of 3 mm was imaged in different wavelength regions ranging from 800-1300 nm. The fluorescence images showed that the scattering effect of light caused by the tissue phantom was suppressed by extending into the longer NIR-II spectral region (Figure 3C). The FWHM analysis of the tube at a depth of 3 mm from the intralipid^®^ surface measured 1487.1±20.6 μm in the NIR-I window, much higher than that measured at wavelength regions above 900-, 1000-, 1100-, 1200- and 1300-nm (562±34.7 μm, 451.5±37.8 μm, 421.5±8.3 μm, 342.5±4.4 μm, 326.7±4.4 μm, respectively) (Figure 3E).

The similar experiment was also performed using ICG-water solution, the result of which showed ICG-water solution emitted less fluorescence signal compared to ICG-rat bile solution under the same experiment condition. The measured width of capillary tube was not obviously broadened with the increase of penetration depth in the NIR-II window, the scattering effect of light caused by Intralipid^®^ solution was significantly suppressed by extending into deeper NIR-II wavelength region (Figure S6A-E). No NIR-II fluorescence signal could be detected in the fresh rat bile (Figure S6F).

### Dynamic NIR-II fluorescence cholangiography and quantitative measurement in a rat model

**Figure 4:**
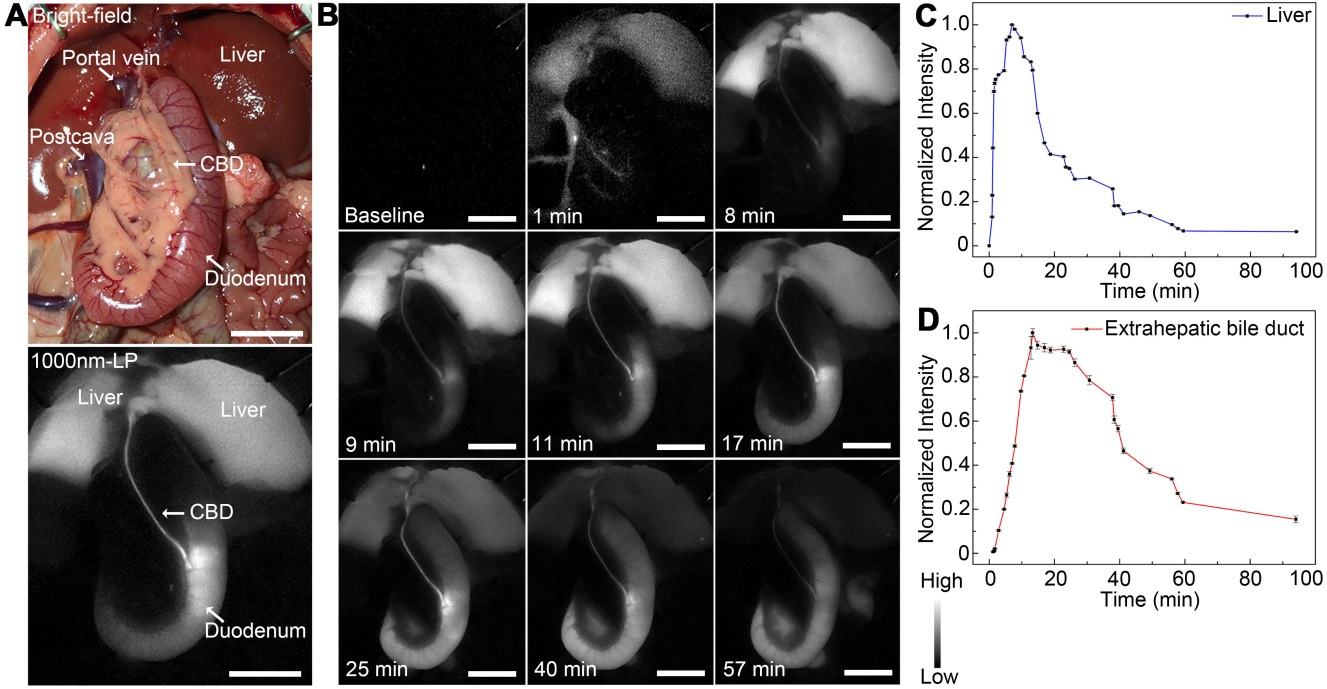
**Dynamic NIR-II fluorescence cholangiography and quantitative measurement in a rat model.** (A) Bright field (upper) and NIR-II fluorescence (lower) images of normal extrahepatic bile duct anatomy of a rat. (B) Continuous dynamic fluorescence imaging from 0 to 94 minutes after intravenous injection of ICG. (C) Dynamic fluorescence intensity measurement of left liver lobe from 0 to 94 minutes after intravenous injection of ICG. (D) Dynamic fluorescence intensity measurement of the extrahepatic bile duct from 0 to 94 minutes after intravenous injection of ICG. Scale bars: A, B, 1.0 cm.

As shown in Figure 4A, the extrahepatic biliary tract could be clearly visualized under the NIR-II detector with a 1000-nm long-pass filter, which was also consistent with the extrahepatic bile duct structures with bright-field imaging. The dynamic fluorescence imaging was monitored in the NIR-II window (Figure 4B). When the fluorescence contrast agent was injected into the vein, the liver became visible in a few seconds, the fluorescent intensity increased rapidly, peaked at about 7 minutes post-injection, and then gradually attenuated to the baseline level about 94 minutes after administration (Figure 4C). For the extrahepatic bile duct, once the ICG was excreted into the bile, the biliary system became gradually visible. About 9 minutes after injection, the integrated extrahepatic biliary tract structure was clearly visible. The fluorescence intensity began to decrease gradually about 13 minutes post-injection and became quite indistinguishable about 94 minutes after administration (Figure 4D). The dynamic NIR-II fluorescence cholangiography and quantitative measurement were also performed in a mouse model, as is shown in Figure S7. The high background signal due to the rapid accumulation of ICG in the liver was reduced in the NIR-II window, allowing the visualization of the hilar bile duct structures more prominently and accurately. The dynamic process of extrahepatic bile duct peristalsis as well as bile excretion from biliary tract into the duodenum were monitored under the NIR-II detector with high spatiotemporal resolution (Movie S1).

### Identification of detailed biliary tract anatomy structure in NIR-II window in a rat model

**Figure 5:**
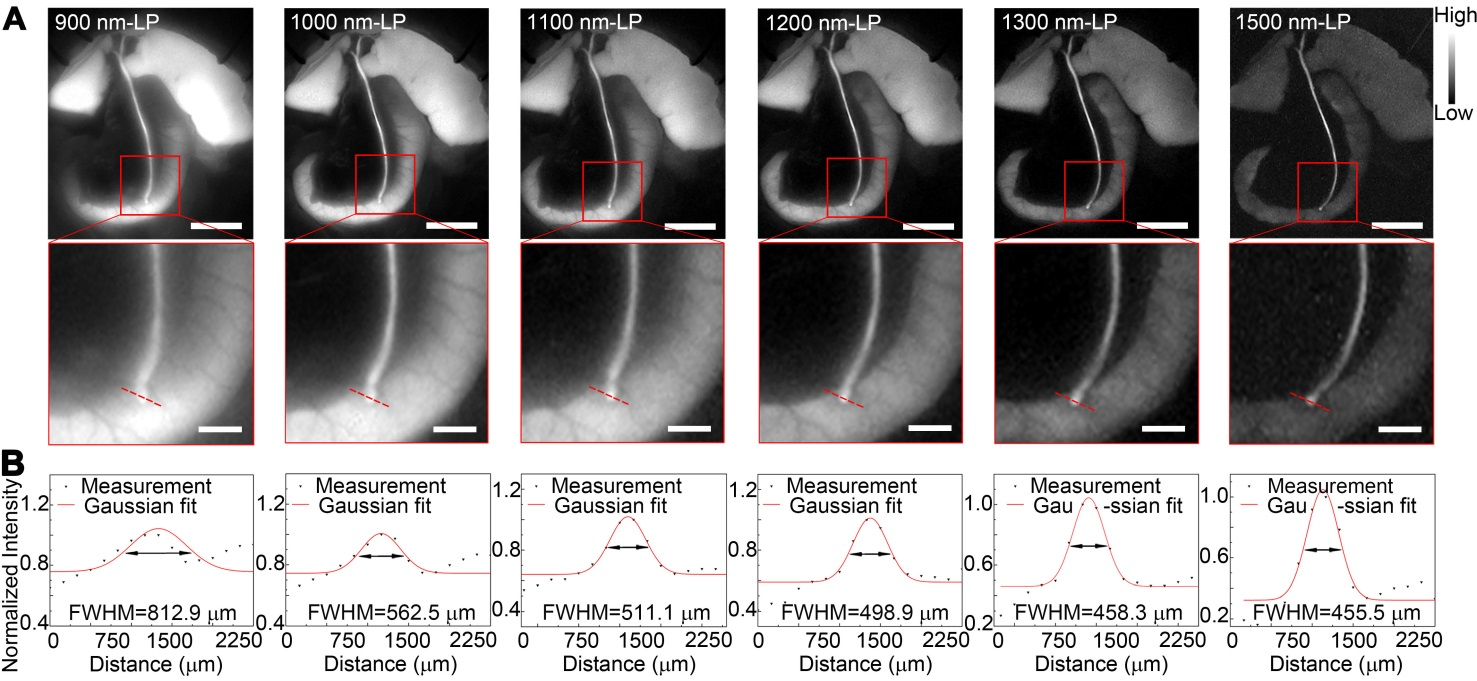
**Identification of detailed biliary tract anatomy structure in NIR-II window in a rat model.** (A) The anatomy structure of the common bile duct joining into duodenum was imaged in the NIR-II window with 900-, 1000-, 1100-, 1200-, 1300-, and 1500-nm long-pass filters in a rat model. (B) Cross-sectional fluorescence intensity profiles along the red dashed bars of the common bile duct. The Gaussian fit to the profile is shown as the red line in each image. Scale bars: A, upper, 1 cm; A, lower, 0.25 cm.

Benefiting from the reduced tissue scattering effect and endogenous autofluorescence, NIR-II imaging might enable the resolution of finer anatomical structures. In this study, the fluorescence images of the extrahepatic bile duct in a rat model were captured in the NIR-II window sequentially using 900-, 1000-, 1100-, 1200-, 1300-, or 1500-nm long-pass filters. As displayed in Figure 5A, the background signal around the extrahepatic bile duct decreased significantly when imaging was performed in longer wavelength regions. Interestingly, by extending into longer NIR-II spectral region, the strong fluorescence signal from ICG metabolites, which had been excreted into duodenum, was filtered out. The structure hidden in the duodenum wall, where the common bile duct merges into duodenum, could be clearly visualized with better spatial resolution. Furthermore, the high background due to the accumulation of ICG in liver was also attenuated at longer NIR-II spectral region (1300-, and 1500-nm wavelength), whereas the fluorescence intensity of extrahepatic biliary tract did not decline. The FWHM of a two-term Gaussian fit to the intensity profiles along red dashed bars in each image were calculated as 812.9 μm, 562.5 μm, 511.1 μm, 498.9 μm, 458.3 μm and 455.5 μm (Figure 5B). The imaging of the specific biliary tract structure in the NIR-II window was also successfully performed in a mouse model (Figure S8). Similarly, by extending into longer NIR-II spectral window, the site where the common bile duct joined into duodenum could be visualized with higher resolution.

### *In vivo* sub-surface extrahepatic biliary tract imaging in NIR-I and NIR-II windows in a rat model

**Figure 6:**
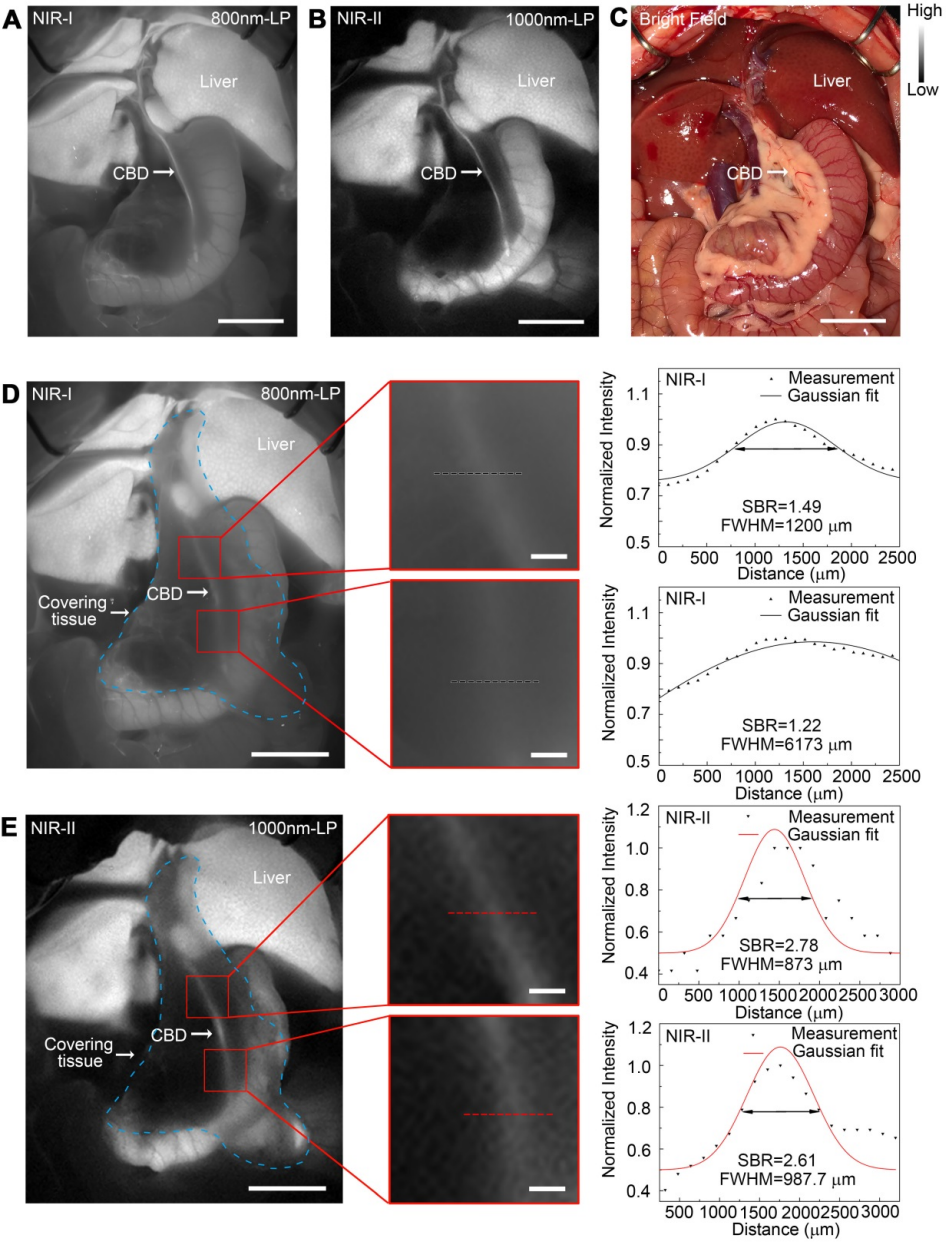
***In vivo* sub-surface extrahepatic biliary tract imaging in NIR-I and NIR-II windows in a rat model.** Representative fluorescence images of the extrahepatic biliary tract in a rat model in (A) NIR-I window and (B) NIR-II window. (C) Corresponding bright-field imaging. NIR-I (D, left) and NIR-II (E, left) fluorescence images of extrahepatic bile duct taken with tissue covered superficially. Bile duct widths (D, right; E, right) were calculated by measuring the FWHM of a two-term Gaussian fit to the intensity profiles along with black dashed bars and red dashed bars. Scale bars: A, B, C, D left, E left, 1 cm; D middle, E middle, 1 mm.

Images were first acquired in a rat model before covering the tissue in the region of interest. For fluorescence imaging performed in the NIR-I window, though the extrahepatic biliary tract could be visualized, there was a higher background around the structures (Figure 6A). Switching to the NIR-II window with a 1000 long-pass filter, a significantly reduced background signal and improved spatial resolution were observed (Figure 6B). The differences between NIR-I and NIR-II imaging in visualizing extrahepatic bile duct structures became significant after broadening the penetrating distance. In the NIR-I region, only proximal part of the common bile duct could be resolved through the tissue with much lower contrast, while the distal common bile duct was hardly visualized (Figure 6D). Switching to the NIR-II window, the imaging exhibited improved contrast and reduced noisy signal, allowing visualization of the distal part of the common bile duct through the covering tissue (Figure 6E). The FWHM of a two-term Gaussian fit to the intensity profiles along black dashed bars in Figure 6D and red dashed bars in Figure 6E were calculated as 1200 μm and 6173 μm, 873 μm and 987.7 μm, respectively. The signal to background ratios (SBRs) defined as the ratios of fluorescence intensity on bile duct to that on the background were calculated as 2.78 and 2.61 (Figure 6E) in the NIR-II window, much higher than those measured in the NIR-I window, 1.49 and 1.22, respectively (Figure 6D). Statistical analyses revealed a significantly higher SBR in the NIR-II window compared to that in the NIR-I window, P < 0.001 (Figure S12A-B).

The comparison of extrahepatic biliary tract imaging in the NIR-I and NIR-II windows was also performed when there existed porcine adipose tissue or thickened inflammatory tissue covering on the region the bile duct went, the result of which showed that the biliary tract structure could be visualized through these tissue components in the NIR-II window, outperforming the imaging performed in the NIR-I window with statistical difference, P < 0.001 (Figure S10 and S11, Figure S12C-D).

The comparison of the sub-surface extrahepatic biliary tract imaging in NIR-I and NIR-II window was also performed in a mouse model (Figure S9). The common bile duct could be visualized even through the covering tissue in the NIR-II window, while in the NIR-I window the biliary tract structure could hardly be resolved through the background noise. Statistical analyses revealed a significantly higher SBR in the NIR-II window compared to that in the NIR-I window, P < 0.001 (Figure S9G).

### Application of NIR-II imaging in identifying acute bile duct stricture in a rat model

**Figure 7:**
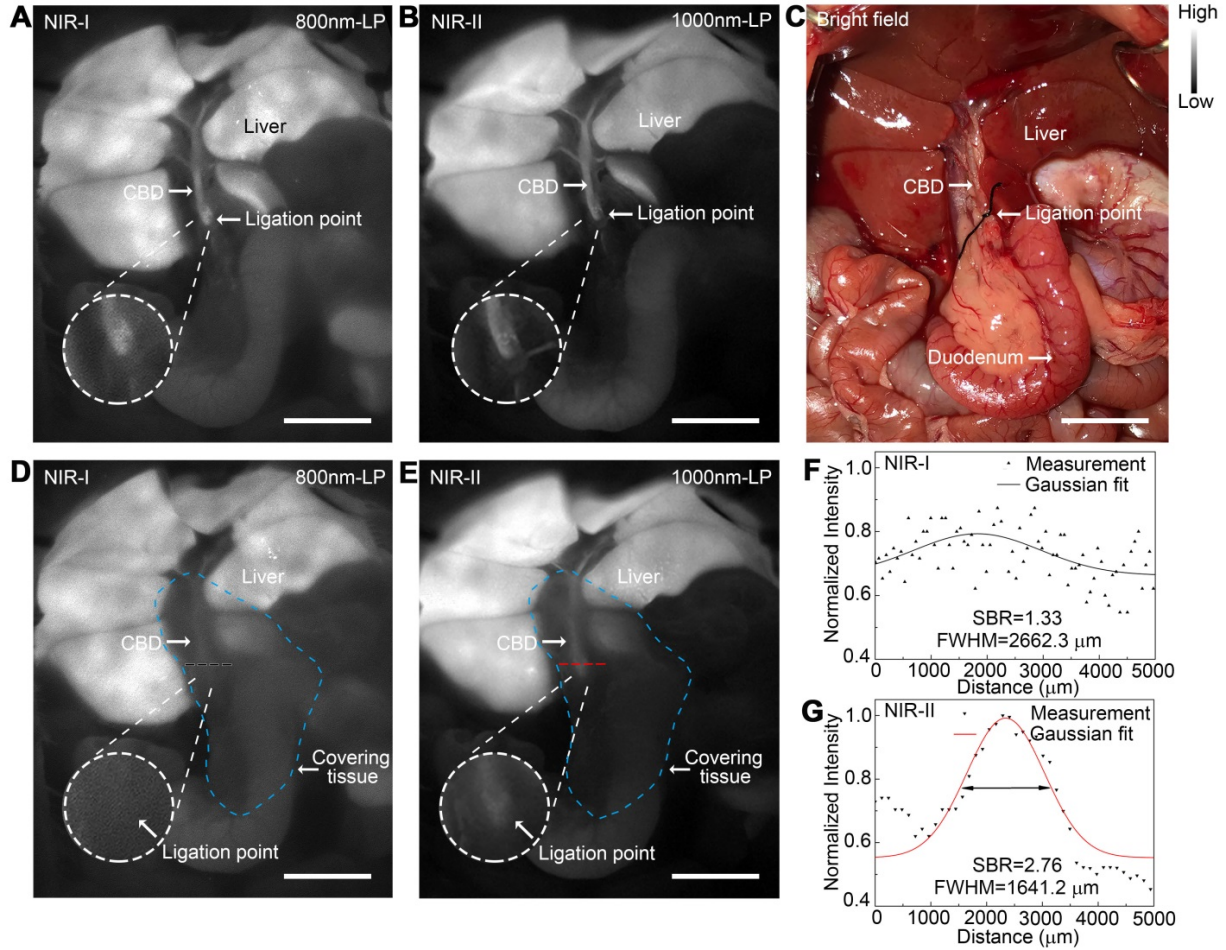
** Application of NIR-II imaging in identifying acute bile duct stricture in a rat model.** Representative fluorescence images of biliary stricture model in (A) NIR-I window and (B) NIR-II window. (C) Corresponding bright-field imaging. NIR-I (D) and NIR-II (E) fluorescence images of acute biliary stricture model taken with tissue covered superficially. (F, G) Bile duct widths were calculated by measuring the FWHM of a two-term Gaussian fit to the intensity profiles along with black dashed bars and red dashed bars. Scale bars: A, B, C, D, E, 1 cm.

An acute bile duct stricture was established in a rat model to identify biliary lesions under NIR-II and NIR-I detectors. As displayed in Figure 7C, the common bile duct was pretreated with ligation using a 5-0 suture before imaging. The ligation point and dilated proximal bile duct were easily identified and imaged with good contrast in the NIR-II window (Figure 7B), outperforming the imaging in the NIR-I window (Figure 7A). Moreover, benefiting from deep penetration depth in the NIR-II window, the ligation site could be resolved even through the covered tissue using 1000-nm long-pass NIR-II detection (Figure 7E), while the specific ligation point could hardly be recognized under NIR-I detector after broadening the penetrating depth (Figure 7D). The intensity across the black dashed bars of interest in the NIR-I window showed insufficient contrast to resolve the precise ligation point from background signal (Figure 7F). Whereas using a 1000-nm long-pass NIR-II detection greatly improved image contrast, allowing the resolution of the precise biliary stricture site (Figure 7G). The FWHM of a two-term Gaussian fit to the intensity profiles along the black dashed bars as well as the red dashed bars were calculated as 2662.3 μm and 1641.2 μm, respectively. The SBRs were calculated as 2.76 in NIR-II window, two times higher than that measured in NIR-I window, 1.33. Statistical analyses revealed a significantly higher SBR in the NIR-II window compared to that in the NIR-I window, P < 0.001 (Figure S12E).

### Application of NIR-II imaging in detecting acute biliary injuries in rat models

**Figure 8:**
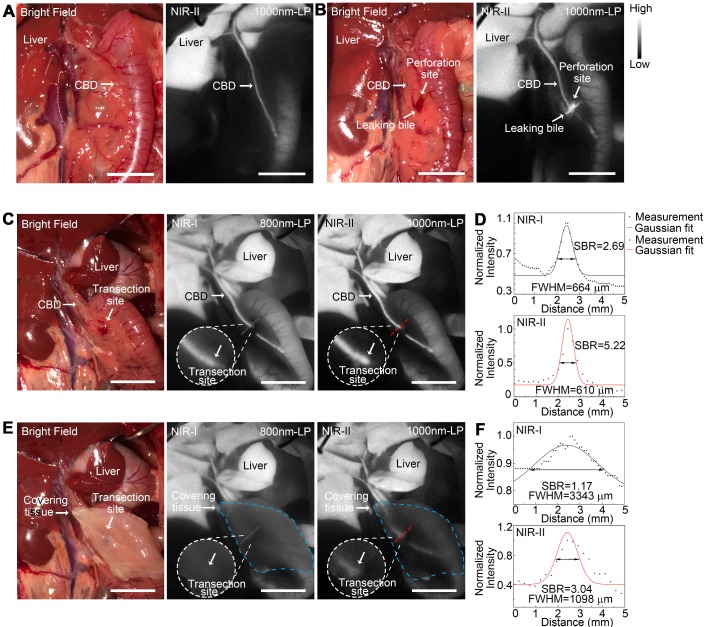
** Application of NIR-II imaging in detecting acute biliary injuries in rat models.** Representative bright field (A, left) and NIR-II fluorescence (A, right) images of normal extrahepatic bile duct in a rat model. Representative bright field (B, left) and NIR-II fluorescence (B, right) images of perforated extrahepatic bile duct in a rat model. NIR-I (C, middle) and NIR-II (C, right) fluorescence image and corresponding bright-field image (C, left) of transected CBD with no tissue covered superficially. (D) Measurement of FWHM of a two-term Gaussian fit to the intensity profiles along with black dashed bars and red dashed bars in Figure C. NIR-I (E, middle) and NIR-II (E, right) fluorescence image and corresponding bright-field image (E, left) of transected CBD with tissue covered superficially. (F) Measurement of FWHM of a two-term Gaussian fit to the intensity profiles along with black dashed bars and red dashed bars in Figure E. Scale bars: A, B, C, E 1 cm.

NIR-II fluorescence imaging of extrahepatic bile duct was firstly performed to confirm that the fluorophore had been continuously excreted into bile (Figure 8A). After intentional common bile duct being perforated, intra-abdominal free bile mixed with blood extravasated, which could be hardly identified with naked eye (Figure 8B, left). Whereas the localization of the perforation site as well as the bile extravasations into the abdominal cavity could be greatly enhanced and visualized using NIR-II imaging (Figure 8B, right).

An acute common bile duct transection injury model was established using electrotome in a rat model (Figure 8C, left). The specific transection site could be well identified in the NIR-II window with higher calculated SBR than that in the NIR-I window (Figure 8C-D). Moreover, the transection site could still be visualized even through the covered tissue in the NIR-II window, while the injury point could hardly be recognized in the NIR-I window after broadening the penetrating depth (Figure 8E-F). The FWHM of a two-term Gaussian fit to the intensity profiles along black dashed bars and red dashed bars in Figure 8C and Figure 8E were calculated as 664 μm and 610 μm, 3344 μm and 1098 μm, respectively. The SBRs were calculated as 2.69 and 1.17 in NIR-I window, 5.22 and 3.04 in NIR-II window, without or with tissue covered on the anatomical region. Statistical analyses revealed a significantly higher SBR in the NIR-II window compared to that in the NIR-I window, P < 0.01 (Figure S12F).

## Discussion

Image guided-surgery is one of the mainstays of the emerging concept of “Precision Medicine and Surgery” 23-25, and aims to provide surgeons an enhanced appreciation of anatomical structures and improve the efficacy and safety of surgical procedures. NIR fluorescence imaging is a novel and promising technique which could provide enhanced visualization of anatomy and organ functions based on the sensitive signal following the specific injection of fluorophores 26-29. Advantages such as optic-based detection, real-time imaging, integratable surgical workflow and reasonable cost provide a rational for its application in various surgical procedures.

Despite many advantages, clinical fluorescence imaging in human patients has been hampered by limited penetration depth in most tissues, which could be attributed to the reason that silicon detectors comprise the majority of cameras in the currently used commercial fluorescence imaging systems 11, the detectable wavelength of which typically ranges between 700-900 nm, hampering deeper tissue imaging during surgery. For cholecystitis patients, obesity and inflammation are quite common, which would result in thickening of the tissue covered on the extrahepatic biliary tract structures to be visualized, making the visualization of the deeper structure or detection of lesion location quite difficult 10. Daskalaki et al. reported that patients with invisible extrahepatic biliary structures with near-infrared fluorescence system more often had cholecystitis than those with visible lesions 30. Similar results were also presented by Dip et al. 31 and Larsen et al. 32.

Recent studies have shown that extending fluorescence imaging into 900-1700 nm wavelength range, also known as NIR-II window, could eliminate the deficiencies of NIR-I imaging 33. The photon-scattering effect in tissues was significantly reduced at longer wavelengths, and the endogenous tissue autofluorescence signal was also weakened, enabling deeper imaging of the underlying structures 34-40. Inspired by these meaningful studies, we applied this novel imaging technique in extrahepatic cholangiography. Our aim was to take advantage of the penetration depth and spatial resolution, which might be of vital importance in improving the NIR fluorescence surgical guidance system and increasing the safety of cholecystectomy, especially in difficult cases.

For successfully performing real-time extrahepatic biliary tract imaging in NIR-II window, the selection of proper contrast agent is crucial. An ideal fluorophore should be FDA-approved yet commercially available with peak emission in NIR-II window 41-50. Though various NIR-II fluorophores with improved quantum yield, photostability and biocompatibility have been developed 51-53, most of them are in preclinical stage, and substantial efforts are needed to translate these preclinical NIR-II dyes into the clinical imaging modality 54.

As a traditional NIR-I fluorescent agent, ICG has been widely used in the clinic, including angiography 55, hepatic funtion testing 56, laparoscopic surgery 57, malignant lymph node tracing 58 and tumor resection guidance 59. However, the fluorescence emission properties of ICG are usally characterized utilizing silicon-based detectors and spectrometers, leading to a sharply decline in the detection ability beyond 900 nm, thus not exploiting the full spectrum of ICG with near-infrared emission.

Recent studies have shown that the emission spectra of aqueous ICG solution, when recorded on a system sensitive to both NIR-I and NIR-II light, could extend well into the NIR-II window even beyond 1500 nm, outperforming the commercial NIR-II fluorophore IE-R1050 60. Considering the ICG metabolism during which it is selectively up-taken by hepatocytes and finally excreted exclusively into bile in its prototype form, we investigated the fluorescence properties of ICG-bile solution in the NIR-II window. Surprisingly, when excreted into bile, the absorption peak of ICG was red shifted and the fluorophore exhibited an enhanced emission value and quantum yield in the NIR-II window compared to its aqueous solution.

By performing the Coomassie Brilliant Blue staining, we provided strong evidence that the fresh bile contained many protein components, which could provide the fluorescent agent with various potential binding sites. As previously illustrated, the absorption spectra of ICG depended on the nature of the solvent and on the concentration of the dye. The rapid shift towards longer wavelengths and higher emission values in the NIR-II window could be attributed to the adsorption of ICG onto the complex bilious compositions including various “plasma type” and “bile-specific” protein ,which could hinder torsional motion and mitigate the triplet state formation of ICG 61, 62, thereby stabilizing the fluorophore and increasing its quantum yield in the NIR-II window. The ICG-bile solution also exhibited excellent photostability, showing higher resistance to the photobleaching effect, which could be beneficial for long-time NIR-II monitoring or bioimaging. Furthermore, results from experiments performed using human bile also demonstrated enhanced fluoresecne emission value of ICG in the NIR-II window, providing a greater clinical translation potential. These results also remind us that the selection of fluorophores for NIR-II imaging may not just limited to agents with peak emission in NIR-II region, the fluorescent agent can also be one with tail emission in the NIR-II window.

We successfully monitored the process of bile excretion into the duodenum as well as bile duct peristalsis within the recommended dose (0.2-0.5 mg/kg) as a potentially novel investigation of biliary dyskinesia 63. More importantly, we used a clinically approved excitation wavelength at a safe power level, which was far below the safe exposure threshold 64. Therefore, with only a spectral tail extending into the NIR-II region, sufficient NIR-II emission of ICG-bile solution could be detected by the high-performance InGaAs camera for real-time extrahepatic cholangiography, without increasing the dye concentration or the excitation power level.

The high background signal hinders the visualization of fine anatomical structures. Here, by extending into the deep NIR-II window, the interfering background around extrahepatic biliary tract significantly reduced, enabling visualization of the fine anatomic structure where the common bile duct joins into duodenum. Interestingly, while using a 1500-nm long-pass emission filter, the strong background due to the rapid accumulation of ICG in the liver was significantly filtered out, yet the fluorescence intensity of extrahepatic biliary tract remained intense enough to be recognized. Since the strong background in liver has always been a problem for the surgeons during cholangiography surgery 57, 65, imaging in the deeper NIR-II window could be a promising option to consider.

We also performed *in vivo* imaging in different animal models to highlight advantages of NIR-II imaging over conventional NIR-I imaging in deeper penetrating ability. In this study, we chose wavelength range above 1000-nm as the NIR-II emission collection region during *in vivo* imaging which allowed both considerable fluorescence signal and deep tissue penetration capability. We excised an intact abdominal tissue from a mouse to simulate the real penetration depth, and with similar tissue components, the mimics could be more reasonable and convincing. NIR-II imaging with the longer wavelength allowed larger penetration depth, making it possible to visualize biliary tract structures in deeper fields.

Bile duct injuries are also common during biliary tract surgical procedures which could lead to serious complications 1, 10. Timely diagnosis and intraoperative management are of vital importance. In this study, we established both acute bile duct stricture rat model and acute bile duct injury rat model to imitate the real circumstance during surgery and utilized the NIR-II imaging technique to detect the precise location of the bile duct lesion through the covered tissue. This technique outperformed the NIR-I imaging, demonstrating the advantages of NIR-II imaging with higher resolution and deeper penetrating ability. These advantages of NIR-II imaging are of vital importance in fluorescence-guided surgery, which could possibly overcome the inherent deficiency of conventional NIR-I light and greatly improve the operation quality.

## Conclusion

The commercially available and clinically approved agent ICG exhibits remarkable NIR-II emission when excreted into bile and could be an excellent fluorophore for real-time NIR-II bile duct imaging. Fluorescence cholangiography in the NIR-II window allows adequate visualization of biliary tract structures with increased penetration depth and high resolution, and might be considered as a valid option for safe cholecystectomy in case of inflammation or obesity, surpassing the performance of the NIR-I window. By switching the detection of traditional silicon-based cameras to emerging indium gallium arsenide-based cameras could promisingly improve the fluorescence surgical navigation system and ensure safe biliary operation, accelerating the clinical translation of NIR-II imaging technology.

## Abbreviations

IOC: intraoperative cholangiography
NIR: near-infrared
ICG: indocyanine green
FDA: Food and Drug Administration
CCD: charge-coupled device
CMOS: complementary metal-oxide-semiconductor
InGaAs: indium gallium arsenide
QY: quantum yield
FWHM: full-width-half-maximum
SBR: signal to background ratio
PBS: phosphate buffered saline
DI: deionized
SDS-PAGE: sodium dodecyl sulfate-polyacrylamide gel electrophoresis
CBD: common bile duct
ICR: Institute of Cancer Research
SD: Sprague-Dawley
kDa: kilodalton
IHC: immunohistochemistry
H&E: hematoxylin-eosin
