# Supplementary material for: Extrahepatic cholangiography in near-infrared II window with the clinically approved fluorescence agent indocyanine green: a promising imaging technology for intraoperative diagnosis
Source: Theranostics. 2020 Feb 19;10(8):3636–51. doi: 10.7150/thno.41127 (PMC7069080; doi:10.7150/thno.41127)
Supplement: Supplementary file 1 — Supplementary figures. [file thnov10p3636s1.pdf]

Supporting Information

Materials and methods

Fresh rat bile collection

After anesthetized, the healthy Sprague-Dawley rat (8 weeks old, female) was secured to a platform in the supine position. Laparotomy was performed and surgical field of interest was fully exposed (Figure S2A). Then the distal part of the common bile duct was carefully dissected from the surrounding tissues (Figure S2C) and perforated by using a microsurgical scissor. A mouse carotid arterial catheter (Inner diameter = 0.3 mm) (Figure S2B) was carefully inserted into the exposed common bile duct (Figure S2D). The suitable insertion depth should be approximate 3 cm. The yellowish transparent liquid would be drained if the catheter was correctly inserted, then the catheter was properly fixed. It should be mentioned that the catheter should be carefully examined to ensure smooth passage before each bile collection experiment since the obstruction could happen in the reutilized catheter.

Brightness comparison of ICG dilutions in bile and water

ICG was resuspended in the supplied dilution and obtain a 2.5 mg/ml stock solution. Rat bile sample used in the in vitro studies were collected from Sprague-Dawley rats (6-8 weeks old, female). For ICG-rat bile solution, fluorescent agent ICG was intravenously injected through tail vein of the rat at a concentration of 0.5 mg/kg, then the excreted bile was collected continuously for 4 hours. To measure ICG emission brightness in NIR-II window, ICG-rat bile solution or ICG-water solution was diluted in collected bile or DI water to 8 continuous concentration, namely 0.25 μg/ml, 0.5 μg/ml, 1.0 μg/ml, 2.0 μg/ml, 4.0 μg/ml, 9.0 μg/ml, 18 μg/ml and 35 μg/ml. The ICG-bile or water solutions were imaged individually and side by side, using a 793-nm excitation (power intensity, 20 mW/cm^2), and the emission was filtered through a 1000-nm long-pass filter (ThorLabs, USA).

To compare the brightness of ICG-bile solution and ICG-water solution in different wavelengths of NIR-II regions, ICG-rat bile solution and ICG-water solution at a concentration of 35 μg/ml were imaged side by side simultaneously with a 900-, 1000-, 1100-, 1200-, 1300-, 1450-, 1500-nm long-pass filter (ThorLabs, USA), using a 793-nm excitation (power intensity, 20 mW/cm^2). The average emission intensity was calculated from a region of interest within the vial.

The photostability assay

The photostability of ICG-rat bile solution and ICG-water solution were investigated under continuous illumination from a 793 nm laser with a power density of 45 mW/cm^2 for 160 minutes, at a concentration of 35 μg/ml. NIR-II fluorescence images were taken every ten minutes. The average emission intensity was calculated from the region of interest within each vial.

Intralipid® phantom imaging

In vitro testing in an Intralipid® phantom was performed as described previously. A 1% Intralipid® solution was prepared by diluting 20% Intralipid® in DI water. A capillary glass tube (Inner diameter = 0.5 mm) filled with ICG-rat bile solution (31.25 μg/ml) was immersed in the prepared 1% Intralipid® solution, the depth of which ranged from 1-6 mm below the top surface. NIR-I and NIR-II imaging at different depths were

performed. For the capillary tube submerged 3 mm below the surface of 1% Intralipid® solution, images with 900-, 1000-, 1100-, 1200-, 1300-nm long-pass filter (ThorLabs, USA) were also taken for further analyses, using a 793-nm excitation (power intensity, 10 mW/cm^2).

Coomassie Brilliant Blue staining assay

Coomassie Brilliant Blue staining was performed according to the standard protocol as previously described. Briefly, fresh bile was collected and protein concentration was determined using Thermo Scientific Pierce BCA Protein Assay Kit. Bile samples (60 μg per well) were separated by 10% sodium dodecyl sulfate-polyacrylamide (SDS-PAGE) gel electrophoresis. The gels were carefully removed and immersed in distilled water, heating to 100℃ for 30 seconds. Then the gels were immersed in prepared staining buffer and heated to 100℃ for 30-60 seconds. After washing the stained gels with cold DI water, distilled water was added and heated to 100℃ for 30-60 seconds. Lastly, repeating washing step several times and acquiring gel images by Bio-Rad ChemiDoc XRS+ System (1708265, California, USA).

Animal experiments

All the animal experiments in this work were conducted strictly in compliance with the requirements and guidelines of the Institutional Ethical Committee of Animal Experimentation of Zhejiang University. Institute of Cancer Research (ICR) mice (6-8 weeks old, female) and Sprague-Dawley (SD) rats (6-8 weeks old, female) were provided from the SLAC laboratory Animal Corporation. (Shanghai, China) and housed in the Laboratory Animal Center of Zhejiang University (Hangzhou, China). The animal housing area was maintained at 24℃ with a 12 hours light/dark cycle, with free access to water and food.

Before each imaging experiment, mice or rat was anesthetized via intraperitoneal injection of 2% pentobarbital (40-50 mg/kg), sufficient depth of anesthesia was maintained, redosing with one-fifth the original dose of pentobarbital only as necessary. The animal was secured to a platform in the supine position, with the ventral side facing the camera and laser source. Laparotomy was performed before imaging, fully exposing the surgical field of interest. Fluorescent agent ICG at a concentration of 0.5 mg/kg was intravenously injected through tail vein before imaging, using a 793-nm excitation. For dynamic NIR-II fluorescence cholangiography, lower dose of fluorophore (0.025 mg/kg) was injected via the inferior vena cava before imaging in order to obtain complete fluorescence curve of extrahepatic biliary tract.

Acute peritonitis mouse model establishment

The method to establish experimental peritonitis model using glacial acetic acid has previously been reported [1, 2]. Adult female mice were randomly divided into two groups (n = 3/group). Mice of experimental group were intraperitoneally injected with 0.15 ml of 2% glacial acetic acid in normal saline. Animals of the control group were given the same volume of normal saline. The general state of health was observed. At 24 hours post glacial acetic acid or saline administration, the animals were anesthetized for collection of blood from eye socket, after which the mice were sacrificed by cervical dislocation and the abdominal tissue were removed for the following experiment. The proportion of neutrophils in the collected blood sample were analyzed by flow

cytometry. Moreover, the histopathological changes by H&E (hematoxylin-eosin) staining and CD45 expression analysis (a cell surface antigen specially expressed in neutrophils) in the generated inflammatory tissue and normal tissue were examined for confirmation of establishment of acute peritonitis model.

Human bile collection

The human bile samples used in this study were obtained from three patients with permission whose bile had been continuously drained for at least 5 days after laparoscopic common bile duct exploration. The study had been approved by the Clinical Research Ethics Committee of Sir Run Run Shaw Hospital of Zhejiang University.

Supplementary figures

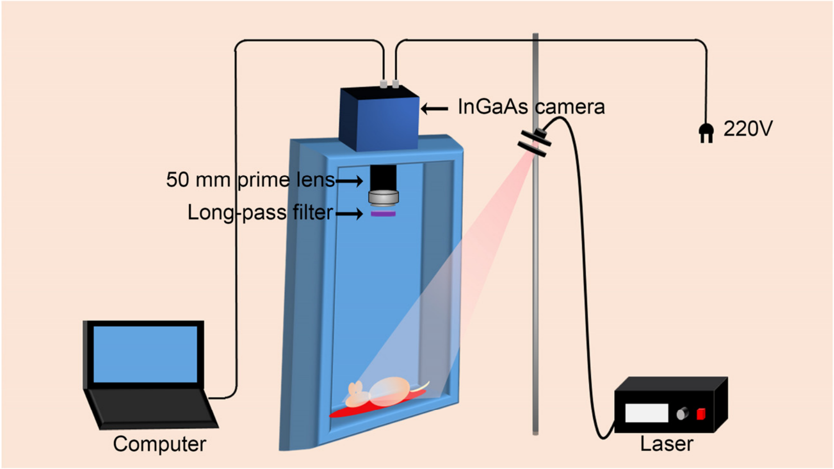

Figure S1 Schematic illustration for NIR-II fluorescence macroscopic imaging system.

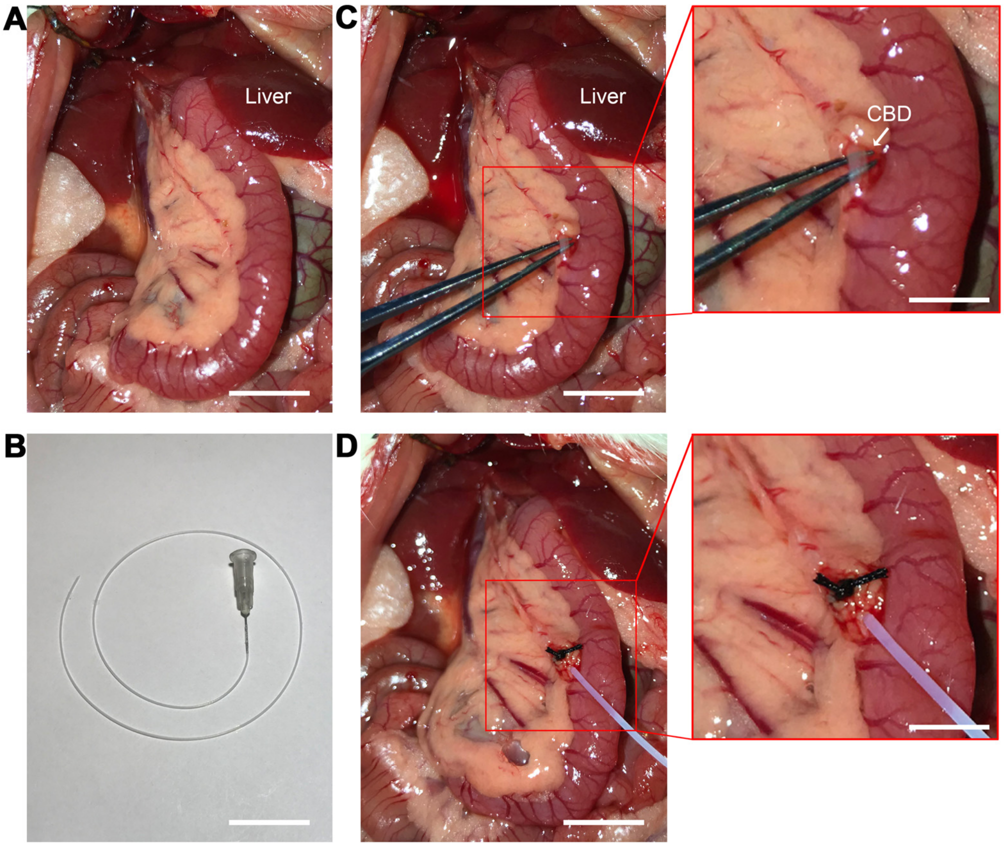

Figure S2 Fresh rat bile collection.

(A) Bright-field imaging of extrahepatic biliary tract of a rat. (B) Imaging of mouse carotid arterial catheter (Inner diameter = 0.3 mm). (C) The dissection of the distal part of common bile duct from the surrounding tissues. (D) The insertion and fixation of catheter to the distal part of common bile duct. Scale bars: A, C left, D left, 1 cm; B, 2 cm; C right, D right, 0.5 cm.

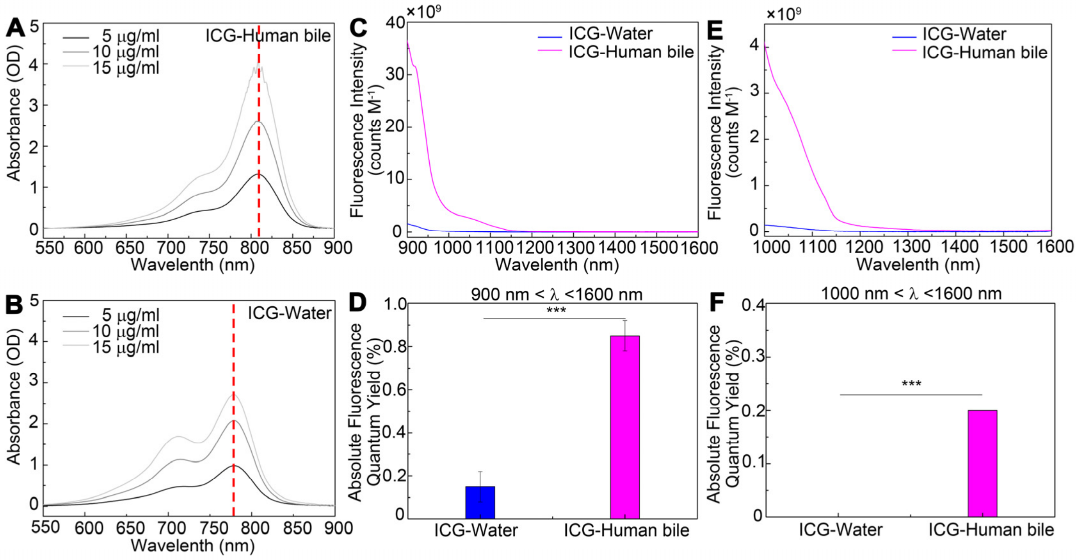

Figure S3 NIR-II emission of ICG-human bile solution.

Absorbance spectra of (A) ICG-human bile solution and (B) ICG-water solution in 550-900 nm wavelength range at concentration of 5, 10, 15 µg/ml. Red dashed bars in each figure shows the absorbance peak. Fluorescence emission profile of ICG in human bile (magenta line) and water (blue line) normalized to equimolar concentration between (C) 900 and 1600 nm wavelength region, (E) 1000 and 1600 nm wavelength region. The absolute fluorescence quantum yield measurement of ICG-water solution (blue column) and ICG-human bile solution (magenta column) between (D) 900 and 1600 nm wavelength region, (F) 1000 and 1600 nm wavelength region.

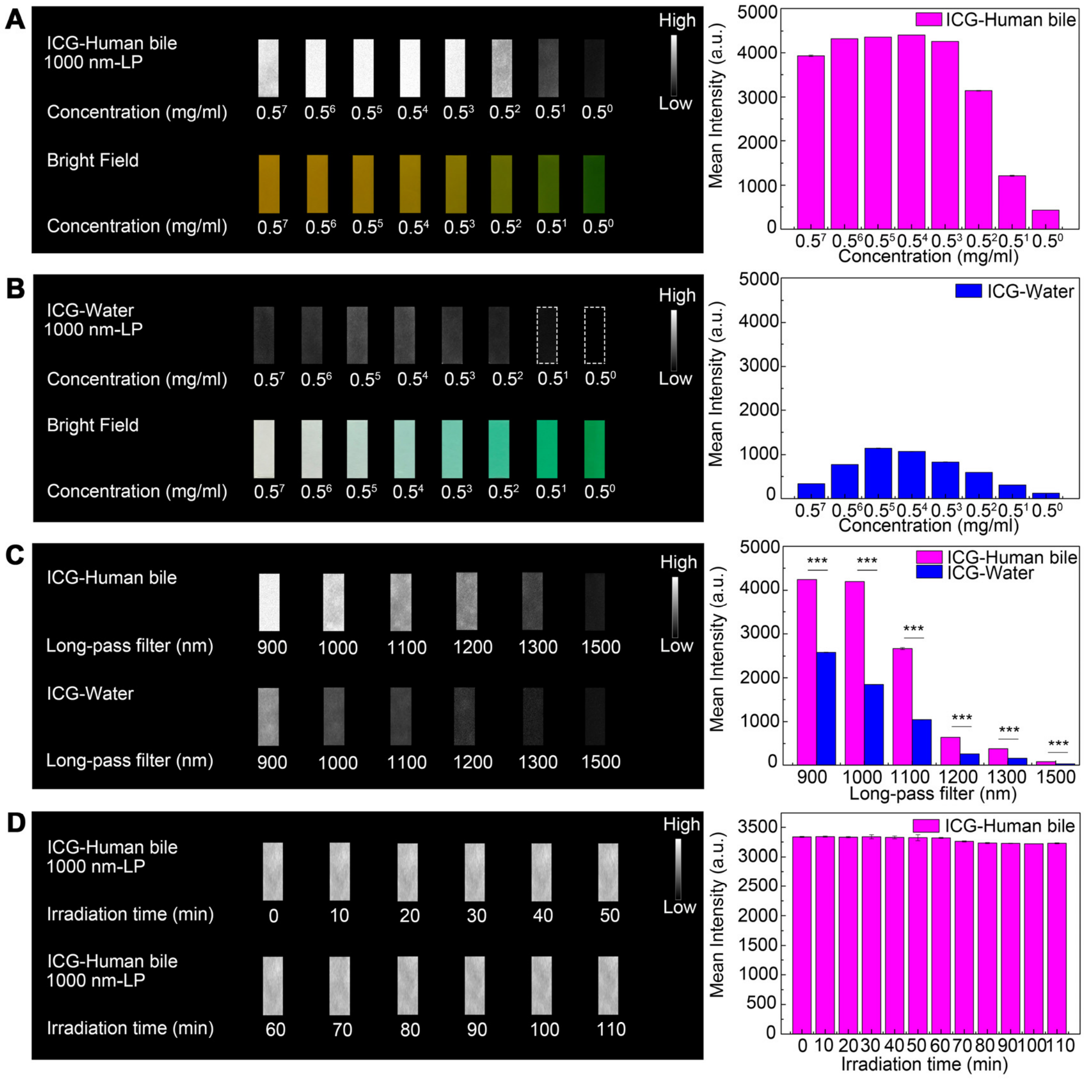

Figure S4 Comparison of optical properties of ICG-human bile and ICG-water solution in NIR-II window.

NIR-II fluorescence and bright field images of vials filled with (A, left) ICG-human bile solution and (B, left) ICG-water solution at 8 different concentrations. Variations of ICG fluorescence intensity measurement as a function of its concentration (mg/ml) in (A, right) human bile and (B, right) water. (C, left) NIR-II fluorescence images of vials filled with ICG-human bile solution and ICG-water solution at different

wavelength regions. (C, right) Fluorescence intensity measurement of ICG-human bile at concentration of 15.625 μg/ml with 900-, 1000-, 1100-, 1200-, 1300-, 1500-nm long-pass filter, using a 793-nm excitation. (D, left) The photostability of ICG-human bile solution under the excitation of a 793-nm laser. Insets are corresponding NIR-II fluorescence photos of ICG-human bile solution at each time point during laser illumination, power density, 45 mw/cm^2. (D, right) NIR-II fluorescence intensity measurement of ICG-human solution at each time point during laser illumination.

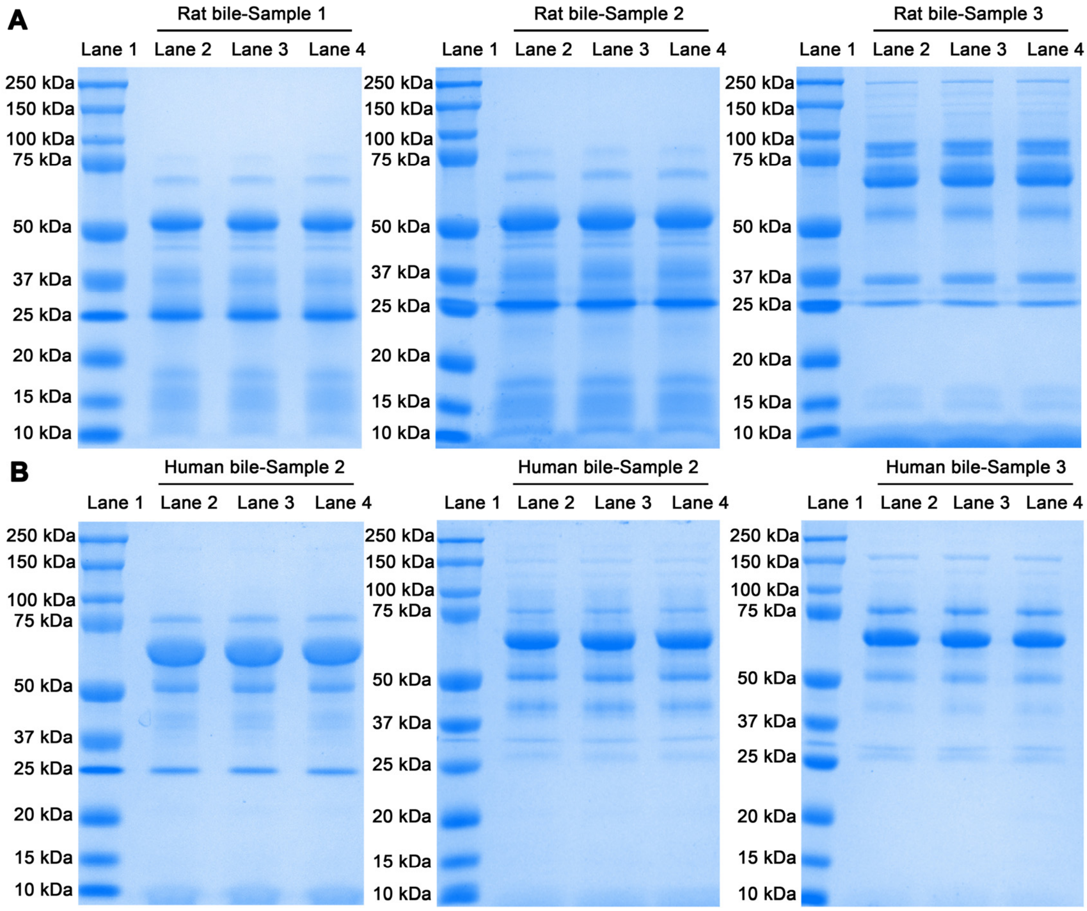

Figure S5 Coomassie Brilliant Blue staining on bile.

Analysis of (A) rat bile and (B) human bile protein components on a 10% SDS-PAGE gel stained by Coomassie Brilliant Blue. Lanes 1 in each gel shows broad range molecular weight standards. Lane 2, 3, 4 in each gel show the staining results in three replicates.

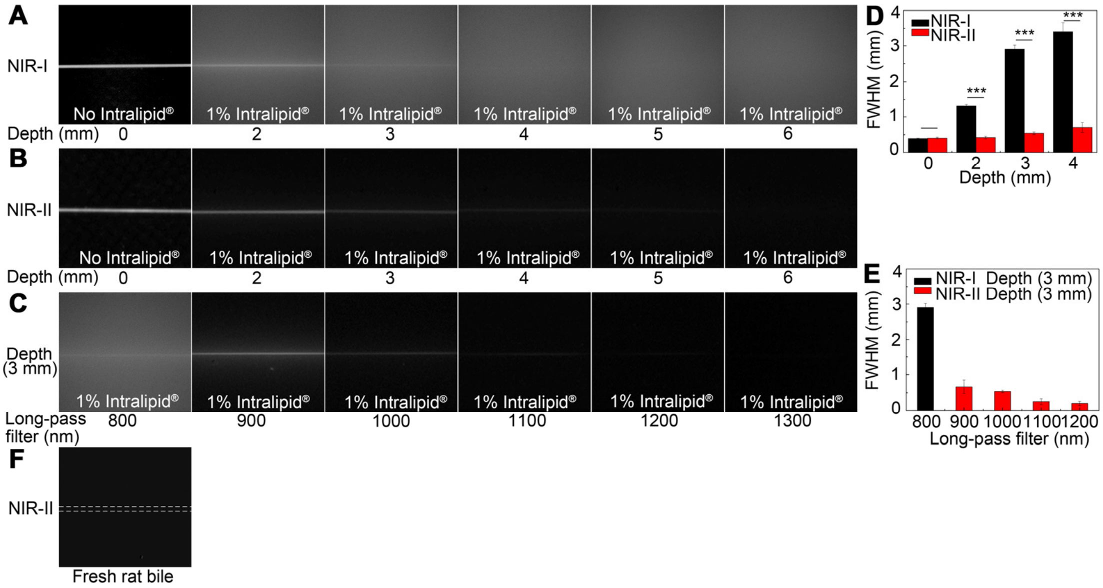

Figure S6 Intralipid® phantom study of ICG-water solution in NIR-I and NIR-II windows.

Fluorescence images in (A) NIR-I window and (B) NIR-II windows of glass capillary filled with ICG-water solution (31.25 µg/ml) at depths of 0, 2, 3, 4, 5 and 6 mm in 1% Intralipid® solution. (C) The capillary filled with ICG-water solution was submerged in 3 mm of 1% intralipid® solution and imaged again with 800-nm long-pass NIR-I detector and 900-, 1000-, 1100-, 1200-, 1300-nm long-pass NIR-II detector. FWHM was calculated for capillary glass tube filled with ICG-water solution (D) at varying depths of 1% Intralipid® solution or (E) with different long-pass filters at the same depth of 1% Intralipid® solution. (F) Capillary tube filled with fresh rat bile in the NIR-II window.

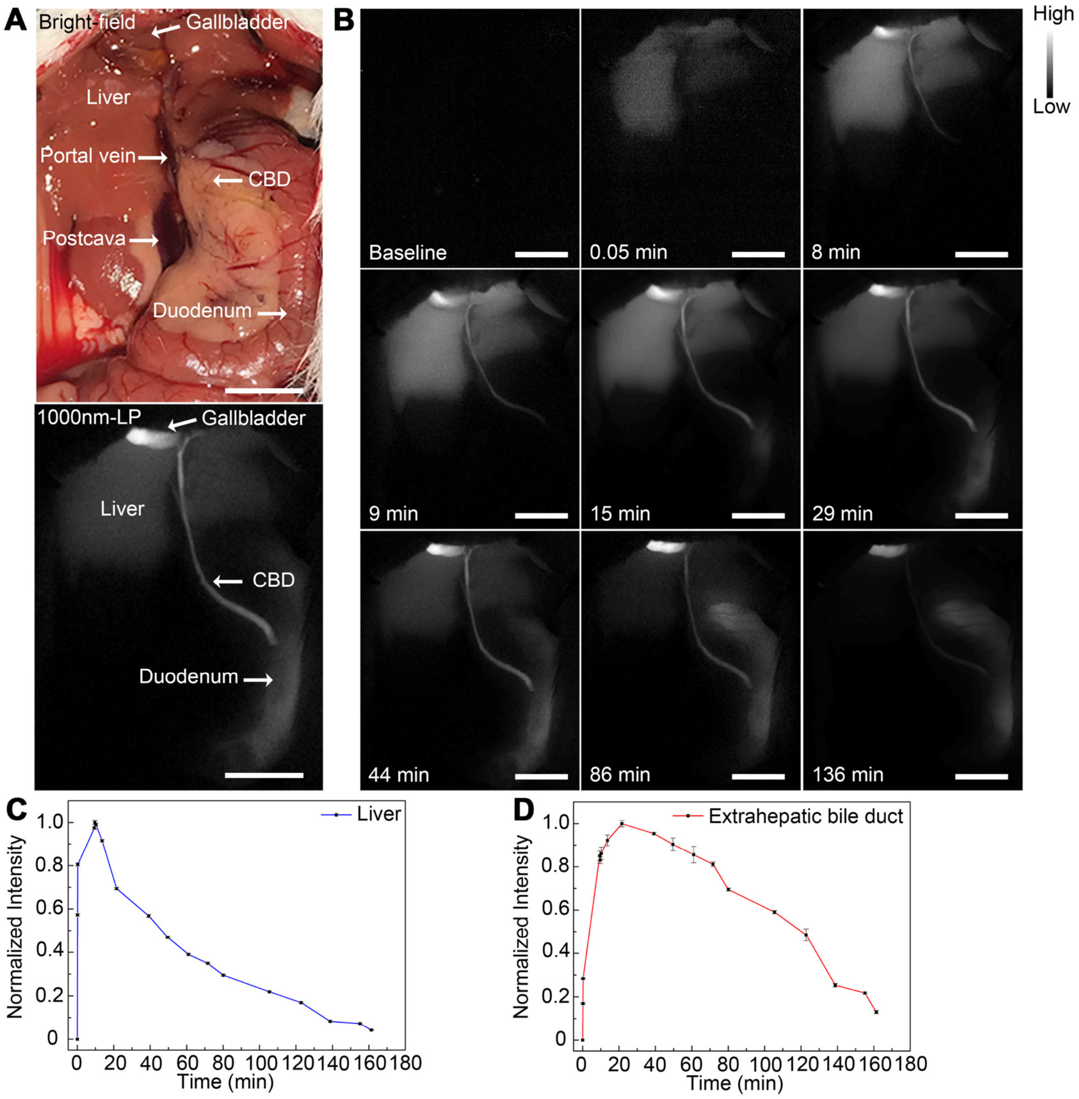

Figure S7 Dynamic NIR-II fluorescence cholangiography and quantitative measurement in a mouse model

(A) Bright field (upper) and NIR-II fluorescence images (lower) of normal bile extrahepatic bile duct anatomy of a mouse. (B) Continuous dynamic NIR-II fluorescence imaging from 0 to 161 minutes after intravenous injection of ICG. (C) Dynamic fluorescence intensity measurement of right liver lobe from 0 to 161 minutes after intravenous injection of ICG. (D) Dynamic fluorescence intensity measurement of extrahepatic bile duct from 0 to 161 minutes after intravenous injection of ICG. Scale bars: A, B, 0.5 cm

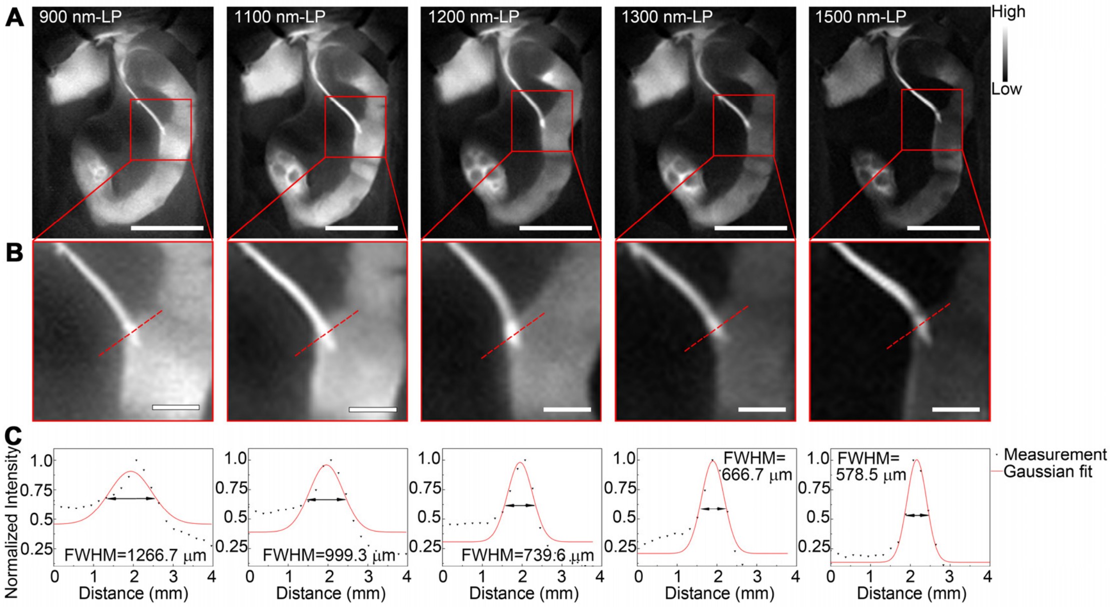

Figure S8 Identification of detailed biliary tract anatomy structure in NIR-II window in a mouse model.

(A-B) The anatomy structure of the common bile duct joining into duodenum was imaged in the NIR-II window with 900-, 1100-, 1200-, 1300-, and 1500-nm long-pass filters in a mouse model. (C) Cross-sectional fluorescence intensity profiles along the red dashed bars of the common bile duct. The Gaussian fit to the profile is shown as the red line in each image. Scale bars: A, 1 cm; B, 2 mm.

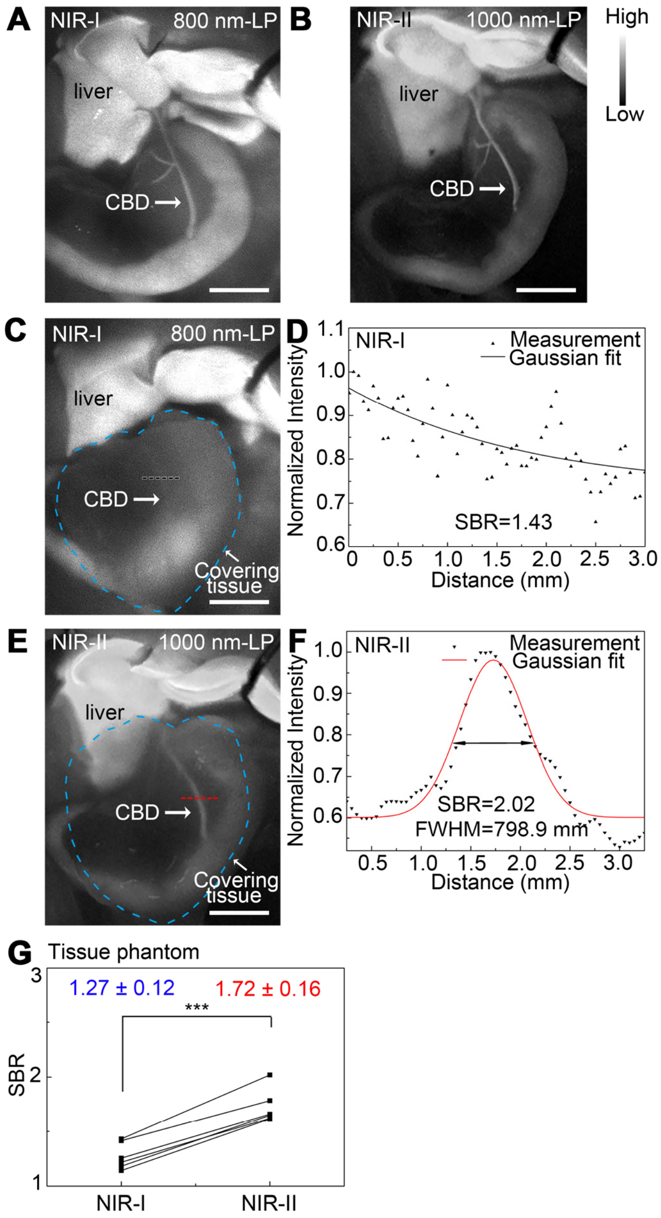

Figure S9 In vivo sub-surface extrahepatic biliary tract imaging in NIR-I and NIR-II window in a mouse model.

Representative fluorescence images of extrahepatic biliary tract in a mouse model in (A) NIR-I window and (B) NIR-II window. NIR-I (C) and NIR-II (E) images of extrahepatic bile duct taken with tissue coved superficially. The intact abdominal tissue was removed from a healthy female ICR mouse. Bile duct width (D, F) was calculated by measuring the FWHM of a two-term Gaussian fit to the intensity profiles along with black dashed bars and red dashed bars. The SBR was defined as the ratio of fluorescence intensity on bile duct to that on the background. (G) Quantitative analysis of the SBR of NIR-I and NIR-II sub-surface bile duct imaging (n = 6). Scale bars: A, B, C, E, 0.5 cm.

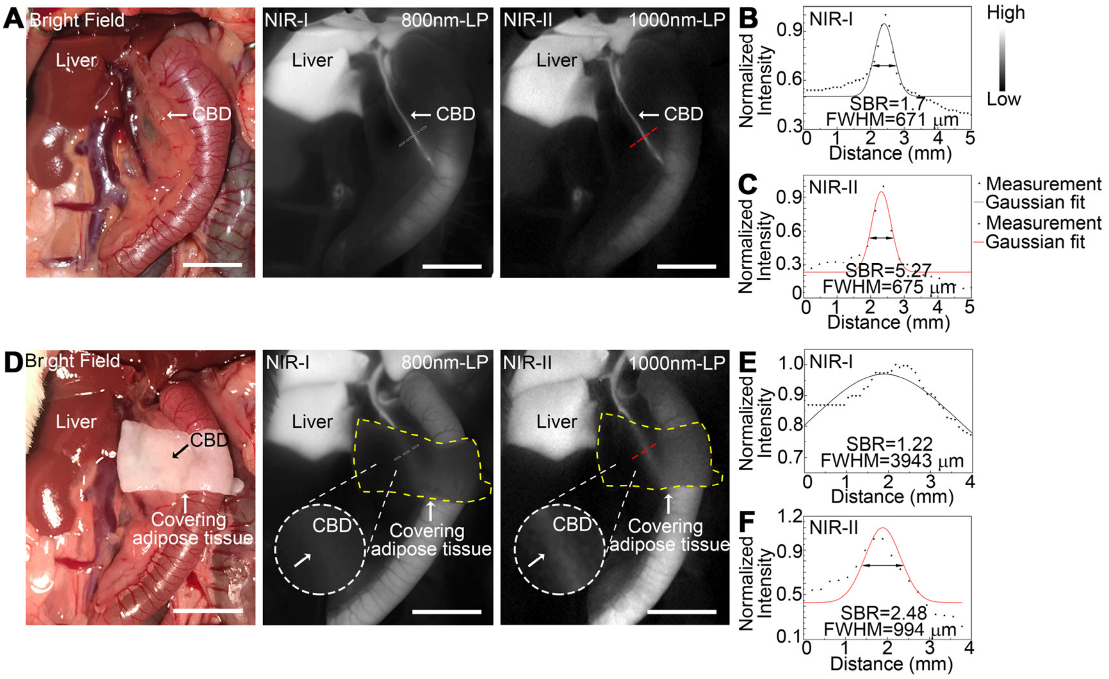

Figure S10 In vivo sub-surface extrahepatic biliary tract imaging in NIR-I and NIR-II windows in established porcine adipose tissue phantom.

Representative fluorescence images of extrahepatic bile duct in a rat model in NIR-I window (A, middle), NIR-II window (A, right) and the corresponding bright-field image (A, left). (B, C) Measurement of FWHM of a two-term Gaussian fit to the intensity profiles along with black dashed bars and red dashed bars in Figure A. NIR-I (D, middle) and NIR-II (D, right) fluorescence image and corresponding bright-field image (D, left) of extrahepatic biliary tract taken with adipose tissue (approximate 1.5 mm thickness) covered superficially. (E, F) Measurement of FWHM of a two-term Gaussian fit to the intensity profiles along with black dashed bars and red dashed bars in Figure D. Scale bars: 1 cm.

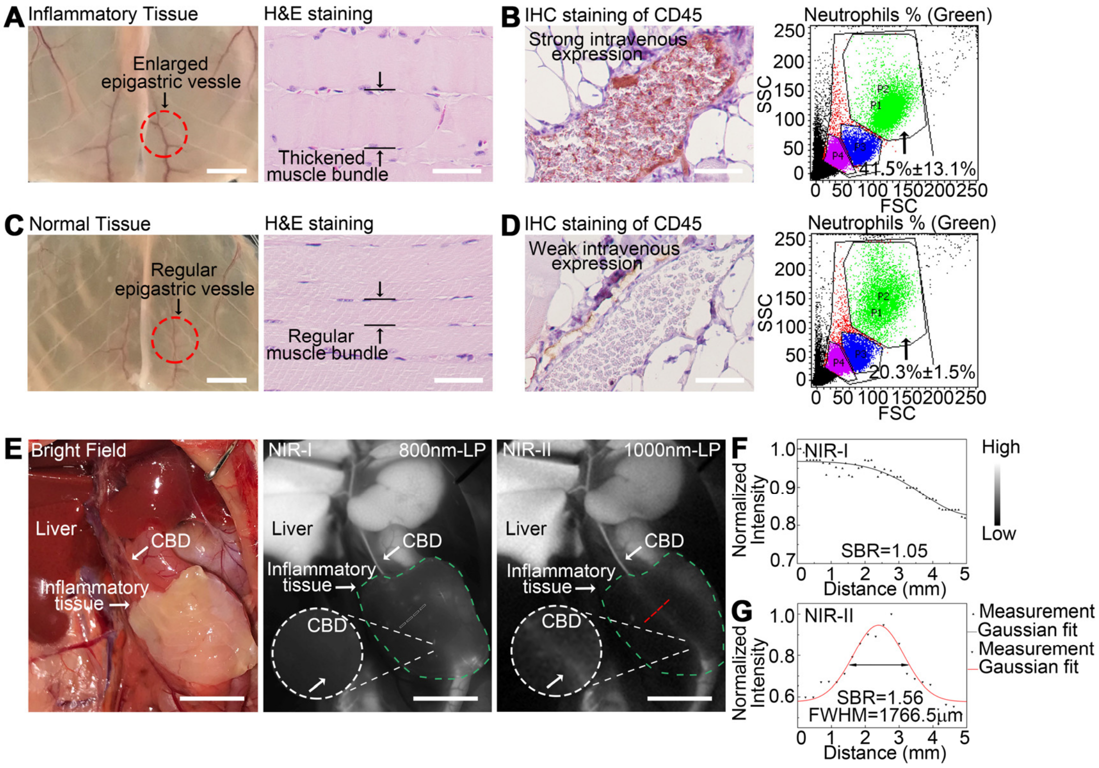

Figure S11 In vivo sub-surface extrahepatic biliary tract imaging in NIR-I and NIR-II windows in established inflammatory tissue phantom.

Establishment of inflammatory tissue. Representative bright field image (A, left) and H&E staining image (A, right) of inflammatory mouse abdominal tissue. (B, left) Expression of CD45 by IHC (immunohistochemistry) analysis in generated inflammatory mouse abdominal tissue. (B, right) Peripheral neutrophils proportion analysis in the established acute peritonitis mouse model by flow cytometry. Representative bright field image (C, left) and H&E staining image (C, right) of normal mouse abdominal tissue. (D, left) Expression of CD45 by IHC analysis in normal mouse abdominal tissue. (D, right) Peripheral neutrophils proportion analysis in normal mouse by flow cytometry.

NIR-I (E, middle) and NIR-II (E, right) fluorescence image and corresponding bright-field image (E, left) of extrahepatic biliary tract taken with inflammatory tissue (approximate 2.0 mm thickness) covered superficially in a rat model. (F, G) Measurement of FWHM of a two-term Gaussian fit to the intensity profiles along with black dashed bars and red dashed bars in Figure E. Scale bars: A left, C left, 0.5 cm; A right, B left, C right, D left, 50 μm; E, 1 cm.

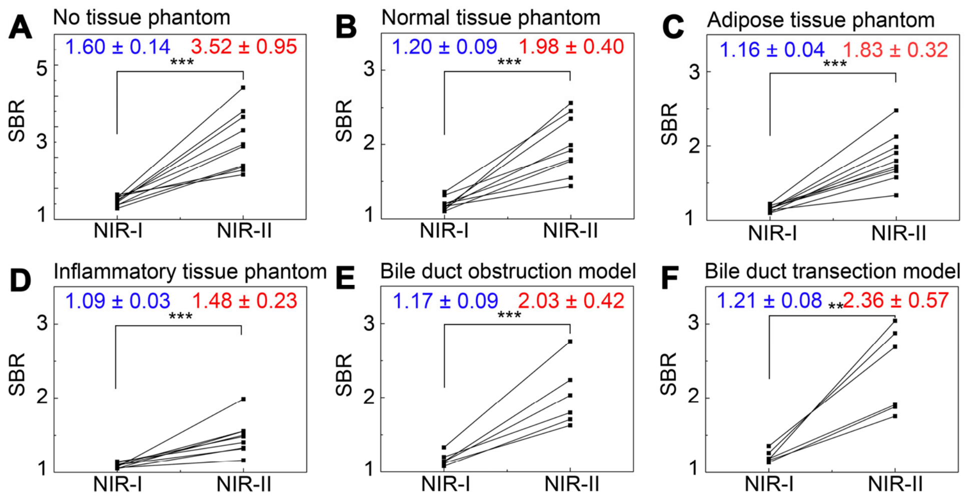

Figure S12 Comparison of SBR for NIR-I and NIR-II cholangiography in rats
Comparison of SBR for NIR-I and NIR-II cholangiography in rats. (A) Quantitative analysis of the SBR of NIR-I and NIR-II cholangiography in no tissue phantom models (n = 10). Quantitative analysis of the SBR of NIR-I and NIR-II sub-surface bile duct imaging in (B) normal tissue phantom models (n = 9), (C) adipose tissue phantom models (n = 10) and (D) acute inflammatory tissue phantom models (n = 9). Quantitative analysis of the SBR of NIR-I and NIR-II sub-surface biliary lesions imaging using normal tissue phantom in (E) acute bile duct obstruction models (n = 6) and (F) acute bile duct transection models (n = 6).

Movie S1 Dynamic imaging of extrahepatic bile duct peristalsis and bile excretion into the duodenum in NIR-II window with a 1000-nm long-pass filter in a mouse model.

References

1. Niu X, Yao H, Li W, Mu Q, Li H, Hu H, et al. delta-Amyrone, a specific inhibitor of cyclooxygenase-2, exhibits anti-inflammatory effects in vitro and in vivo of mice. Int Immunopharmacol. 2014; 21: 112-8.
2. Clementi G, Caruso A, Cutuli VM, Prato A, Mangano NG, Amico-Roxas M. Antiinflammatory activity of adrenomedullin in the acetic acid peritonitis in rats. Life Sci. 1999; 65: Pl203-8.
